# Bystander Effects of Hypoxia-Activated Prodrugs: Agent-Based Modeling Using Three Dimensional Cell Cultures

**DOI:** 10.3389/fphar.2018.01013

**Published:** 2018-09-18

**Authors:** Cho R. Hong, Gib Bogle, Jingli Wang, Kashyap Patel, Frederik B. Pruijn, William R. Wilson, Kevin O. Hicks

**Affiliations:** ^1^Auckland Cancer Society Research Centre, University of Auckland, Auckland, New Zealand; ^2^Auckland Bioengineering Institute, University of Auckland, Auckland, New Zealand; ^3^Maurice Wilkins Centre, University of Auckland, Auckland, New Zealand

**Keywords:** hypoxia-activated prodrugs, agent-based modeling, PKPD models, bystander effects, multicellular spheroids, PR104A, SN30000, nitrogen mustards

## Abstract

Intra-tumor heterogeneity represents a major barrier to anti-cancer therapies. One strategy to minimize this limitation relies on bystander effects via diffusion of cytotoxins from targeted cells. Hypoxia-activated prodrugs (HAPs) have the potential to exploit hypoxia in this way, but robust methods for measuring bystander effects are lacking. The objective of this study is to develop experimental models (monolayer, multilayer, and multicellular spheroid co-cultures) comprising ‘activator’ cells with high expression of prodrug-activating reductases and reductase-deficient ‘target’ cells, and to couple these with agent-based models (ABMs) that describe diffusion and reaction of prodrugs and their active metabolites, and killing probability for each cell. HCT116 cells were engineered as activators by overexpressing P450 oxidoreductase (POR) and as targets by knockout of POR, with fluorescent protein and antibiotic resistance markers to enable their quantitation in co-cultures. We investigated two HAPs with very different pharmacology: SN30000 is metabolized to DNA-breaking free radicals under hypoxia, while the dinitrobenzamide PR104A generates DNA-crosslinking nitrogen mustard metabolites. In anoxic spheroid co-cultures, increasing the proportion of activator cells decreased killing of both activators and targets by SN30000. An ABM parameterized by measuring SN30000 cytotoxicity in monolayers and diffusion-reaction in multilayers accurately predicted SN30000 activity in spheroids, demonstrating the lack of bystander effects and that rapid metabolic consumption of SN30000 inhibited prodrug penetration. In contrast, killing of targets by PR104A in anoxic spheroids was markedly increased by activators, demonstrating that a bystander effect more than compensates any penetration limitation. However, the ABM based on the well-studied hydroxylamine and amine metabolites of PR104A did not fit the cell survival data, indicating a need to reassess its cellular pharmacology. Characterization of extracellular metabolites of PR104A in anoxic cultures identified more stable, lipophilic, activated dichloro mustards with greater tissue diffusion distances. Including these metabolites explicitly in the ABM provided a good description of activator and target cell killing by PR104A in spheroids. This study represents the most direct demonstration of a hypoxic bystander effect for PR104A to date, and demonstrates the power of combining mathematical modeling of pharmacokinetics/pharmacodynamics with multicellular culture models to dissect bystander effects of targeted drug carriers.

## Introduction

The diversity of cellular phenotypes within tumors, driven by profound genetic and microenvironmental heterogeneity, represents a central challenge to cancer therapy. In particular, subsets of cells that do not express, or are not dependent on, a specific therapeutic target frequently limit response and drive relapse (Marusyk et al., 2012; Bedard et al., 2013; Junttila and de Sauvage, 2013; Andor et al., 2016; Maley et al., 2017). A general strategy for minimizing the negative prognostic impact of intra-tumor heterogeneity is to utilize two-stage drug delivery systems in which targeting and effect are decoupled. In the first stage a feature expressed selectively by a biologically important tumor cell population is targeted with a masked therapeutic such as a prodrug, antibody-drug conjugate or drug-loaded nanoparticle. In the second stage, release of a drug with broad-spectrum cytotoxicity provides the potential for local diffusion to eliminate non-targeted cells. This type of ‘bystander effect’ is frequently invoked as a critically important component of the antitumor activity of such agents (Meng et al., 2012; Foehrenbacher et al., 2013a; Cao et al., 2014; Ogitani et al., 2016; Wilson et al., 2007), but direct demonstration of bystander effects in tumor tissue is challenging.

Bystander effects resulting from local diffusion of active drug metabolites are also critically important in overcoming non-uniform delivery of the targeting agent, which is a major limitation with many prodrugs and macromolecular drug delivery systems (Hicks et al., 2003; Minchinton and Tannock, 2006; Kyle et al., 2007; Firer and Gellerman, 2012; Dewhirst and Secomb, 2017). A holistic understanding of the intra-tumor pharmacology of such agents would be greatly advanced by experimentally informed pharmacokinetic/pharmacodynamic (PKPD) models that explicitly address the reaction and diffusion of both the targeting agent and the released active drug in the tumor microenvironment (Shah et al., 2012; Kalra et al., 2014; Singh et al., 2016). We have previously explored steady-state continuum mathematical models in this context, applying these to the description of intratumor concentration gradients and cell killing by hypoxia-activated prodrugs (HAPs) (Hicks et al., 1998, 2006, 2010; Hay et al., 2007; Foehrenbacher et al., 2013a,b). In these studies, Green’s function PKPD models were parameterized using measurements of prodrug/drug reaction, diffusion and cytotoxicity in single cell suspensions and in 3D multicellular layer (MCL) cultures (Hicks et al., 2003), the latter as *in vitro* models for the extravascular compartment in tumors (Hicks et al., 2010; Foehrenbacher et al., 2013a,b). Recently, we have developed agent-based models (ABMs) as more flexible tools for simulation of intratumor PKPD and have applied these to simulating the growth of multicellular spheroids and monolayer cultures, and their response to radiation in combination with the preclinical HAP SN30000 (Mao et al., 2018). These ABM permit explicit description of prodrug/drug concentrations in each cell in a multicellular system, including in genetically heterogeneous cell populations, as well as extracellular concentration gradients, along with cell fate decisions that are influenced by microenvironmental context such as local concentrations of oxygen, nutrients and drugs.

Here, we use an ABM approach as a tool to develop PKPD models that explore bystander effects resulting from metabolic activation of HAPs. We focus on two HAPs that generate cytotoxic metabolites with very different properties. SN30000, an analog of the well-studied HAP tirapazamine with improved ability to penetrate into hypoxic tissue (Hicks et al., 2010), is metabolized to a DNA-reactive oxidizing free radical under hypoxia (Hicks et al., 2010; Anderson et al., 2014). PR104A is a clinical stage (McKeage et al., 2012; Konopleva et al., 2015) dinitrobenzamide HAP with a nitrogen mustard moiety that is activated by reduction to the corresponding hydroxylamine (PR104H) and amine (PR104M) under hypoxia (**Figure 6A**) (Patterson et al., 2007); these active metabolites have sufficient stability to diffuse out of hypoxic cells and are thus candidate mediators of bystander effects (Foehrenbacher et al., 2013a). We quantify bystander effects by testing the ability of cells with a high capacity for HAP metabolism (“activators”) to increase HAP-dependent killing of cells with low metabolic capacity (“targets”) in hypoxic spheroids grown as co-cultures of both cell lines. Activator and target cell lines were generated by manipulating expression of P450 oxidoreductase (POR), which is a major reductase responsible for activation of SN30000 (Hunter et al., 2015) and PR104A (Guise et al., 2007; Su et al., 2013a) in hypoxic tumor cells. We quantify clonogenic cell killing of activators and targets in spheroids and simulate this activity using ABM with measured parameters for tissue diffusion, reaction (metabolism) and cytotoxicity of the HAPs. This integrated experimental and modeling approach demonstrates that bioreductive activation of PR104A (but not SN30000) generates an efficient bystander effect, and enabled us to identify a downstream metabolite of PR104H and PR104M as the main contributor.

## Materials and Methods

### Compounds

SN30000, its 1-oxide and nor-oxide metabolites (Hicks et al., 2010), PR104A (Patterson et al., 2007), and FSL61 (Su et al., 2013b) were synthesized at the Auckland Cancer Society Research Centre (ACSRC). Compounds were dissolved in methanol (PR104A) or DMSO (all others) and stored at -80°C. 2-(Bis(2-chloroethyl)amino)-5-(hydroxyamino)-*N*-(2-hydroxyethyl)-3-nitrobenzamide (Metabolite A in **Figure 6A**) was prepared by reducing the corresponding 3,5-dinitro compound (Cmpd 1 in **Figure 6A**) with zinc powder (30 mg) and ammonium acetate (30 mg) in deoxygenated acetonitrile (1 mL) in an anaerobic chamber (Sheldon Manufacturing Inc., Cornelius, OR, United States). After mixing for 2 min, the solution was filtered and stored at -80°C.

### Cell Lines

HCT116 cells were authenticated by STR profiling. HCT116-derived lines with bi-allelic knockout of *POR* (Hko2; Su et al., 2013a) and with forced expression of an *N*-terminal truncated cytosolic variant of POR (HCT116/POR#6; Foehrenbacher et al., 2013a) were transfected with plasmids to construct POR-null “target” (PORko-R and PORko-G) and high-POR “activator” (POR-R and POR-G) cell lines with red (R) and green (G) fluorescent protein markers and antibiotic selection markers as detailed in **Supplementary Figure 1**. All cells were passaged in α-MEM containing 5% FBS, which was also used in all experiments. The POR-R and POR-G cell lines were passaged in 2 μM puromycin and the PORko-R and PORko-G lines in 1 mg/mL neomycin (G418).

### Flow Cytometry Analysis for POR Activity and Fluorescent Protein Expression

Enzymatic activity of POR was assessed using a fluorogenic probe for one-electron reductases, FSL61, in a flow cytometry assay as previously described (Su et al., 2013b). Expression of fluorescent proteins was monitored using an LSR II flow cytometer (BD Biosciences, San Jose, CA, United States) at 488/533 nm (excitation/emission) for copGFP and EGFP, 488/610 nm for mRuby and 640/675 nm for TagRFP657.

### Confocal Microscopy

Spheroids were imaged using a Zeiss LSM 710 inverted confocal microscope (Carl Zeiss AG, Germany). Fluorescent images were collected at 405/410–590 nm for H33342, 488/661–755 nm for Alexa647, 488/490–578 nm for copGFP and EGFP, 561/582–754 nm for mRuby and 633/582–754 nm for TagRFP657 using an EC Plan-Neofluar 10x/0.30 M27 objective.

### Monolayer Clonogenic Assay Under Anoxia

Log-phase cells were trypsinized and cell pellets, collected by centrifugation, were transferred to a Bactron anaerobic chamber (Sheldon Manufacturing Inc., Cornelius, OR, United States) under 3–5% H_2_/5% CO_2_/balance N_2,_ <100 ppm O_2_, with a palladium/activated carbon catalyst to maintain anoxia. (The term “anoxia” is used throughout to refer to the gas phase, without necessarily implying complete anoxia in monolayers or spheroids as discussed below.) Cells were resuspended and seeded at 10^5^ cells/0.15 mL in 96-well plates using medium and plates equilibrated in the chamber for 3 days to ensure complete deoxygenation. After incubation at 37 °C for 2 h for cell attachment, cells were exposed to drugs for 1 h, removed from the chamber, trypsinized, counted with a Coulter counter (model Z2; Beckman Coulter^TM^, Indianapolis, IN, United States) and plated in 6-well plates. After 10 days, plates were stained with methylene blue (2 g/L in EtOH:H_2_O, 1:1 v/v) and colonies with more than 50 cells were counted. Plating efficiency (PE) was calculated as number of colonies/number of cells plated, and surviving fraction (SF) was determined as PE(drug treated)/PE(control).

### Spheroid Formation

Spheroids were formed in hanging drop cultures using GravityPLUS plates (InSphero AG, Brunswick, ME, United States) according to the manufacturer’s protocol. 1,000 or 3,000 cells/well were seeded and incubated for 3 days without antibiotic selection. Spheroids were then transferred to pre-wet GravityTRAP plates (InSphero AG, Brunswick, ME, United States) and grown for a further day before use.

### Spheroid Clonogenic Assay Under Anoxia

Oxic media in the GravityTRAP plates was replaced with anoxic media (three times) in the anaerobic chamber and spheroids were then transferred to new GravityTRAP plates (one spheroid/well) that had been pre-equilibrated under anoxia for >3 days. Spheroids were incubated for 1 h and residual oxygen was further decreased by replacing with anoxic medium three times. Spheroids were incubated for an additional 1 h before drug treatment in a final volume of 80 μL/well. After 1 h drug exposure, plates were removed from the chamber and groups of four spheroids treated at the same concentration were pooled and trypsinized for clonogenic assay using selective media containing either 2 μM puromycin or 1 mg/mL G418.

### Multicellular Layer (MCL) Diffusion Assay

Multicellular layers were grown for 4 days as described (Pruijn et al., 2005), after seeding support membranes with mixtures of activator (POR-R) and target (PORko-G) cells. MCLs were mounted in a custom diffusion chamber (Hicks et al., 2003) and equilibrated under flowing 5% CO_2_/95% N_2_ for 1 h. SN30000 (100 μM) and ^14^C-urea (1 μM, 2.11 GBq/mmol, Amersham, Australia) were added to the donor compartment. 100 μL medium was sampled from the donor and receiver compartments at intervals for liquid scintillation counting (Packard Tricarb Scintillation, Ramsey, MN, United States) and were stored at -80°C for HPLC analysis.

### HPLC Analysis of SN30000 and Metabolites

Culture medium samples (40 μL) were analyzed without further processing, using an Agilent 1200 Series HPLC with Zorbax Eclipse XDB-C18 column (4.6 mm× 150 mm, 5 μm) at a flow rate of 0.75 mL/min. The mobile phase was linear gradient of 45 mM formate buffer/formic acid (pH 3.5) and 60% acetonitrile/20% methanol/20% water (10–95% organic phase over 19 min) with detection at 252 nm. Analytes were quantified using calibration curves of authentic compounds in each experiment.

### LC-MS/MS Assay for PR104A Metabolites

POR-R cells (10^6^ cells/0.5 mL) in 24-well plates were exposed to 100 μM PR104A under anoxia for 3 h. Plates were centrifuged (216 × *g*, 4°C, 8 min) extracellular medium was removed and proteins precipitated with an equal volume of ice-cold methanol. Cold methanol (100 μL) was then added to each well to extract intracellular PR104A and metabolites. Samples were stored at -80°C, then thawed, centrifuged (13,000 × *g*, 5 min, 4°C), diluted with two volumes of ammonium formate buffer (4.5 mM, pH 4.5) and analyzed with an Agilent LC-MS/MS (model 6460) using an electrospray ionization/atmospheric pressure chemical ionization multimode source and photodiode array absorbance detector as described previously (Patel et al., 2007). Metabolites were identified using MS scans and quantified using calibration curves of PR104A at 254 nm assuming extinction coefficients of metabolites are equal to PR104A as demonstrated for PR104H and PR104M (Singleton et al., 2009).

### ABM Simulations

Two ABMs have been used: an on-lattice 3D model for spheroids, and a model employing a 1D approximation for monolayers. Both models are described fully elsewhere (Mao et al., 2018) and are outlined in **Supplementary Materials**. Each cell is simulated as an autonomous agent with its individual history, phenotype and fate in response to drug exposure and concentrations of nutrients (oxygen and glucose). In the experiments reported here, regular medium replenishment ensured that glucose was never a constraint; therefore the dependence of growth and survival on glucose was turned off in the simulations.

#### Growth Model

The spheroid grows in a specified volume and depth of unstirred medium, containing dissolved oxygen; the whole culture is modeled, rather than the spheroid in isolation, as detailed under Spheroid Growth Model in **Supplementary Materials**. The model reproduces both the volume and depth of medium (typically 0.2 mL and 6.1 mm, respectively). There are two linked solvers, using a coarse grid for the medium and an embedded fine grid for the spheroid. Here, we restrict attention to the fine grid solver, in which cells occupy sites on a regular 3D lattice. The volume of a cubic lattice site is twice that of an average HCT116 cell, resulting in a cell volume fraction of 0.5 as previously determined in HCT116 MCL cultures (Foehrenbacher et al., 2013a). Cells grow in volume at a rate depending on the local oxygen concentration and divide when volume reaches a preset value. When a cell divides the daughter cell occupies an adjacent empty lattice site; if a vacancy does not exist cells are moved radially outward to create one. Oxygen concentration varies spatially and temporally, controlled by cellular consumption and the specified level at the medium-air interface. Cells starved of oxygen eventually undergo cytolysis, leading to the development of central necrosis matching that observed in HCT116 spheroids (Mao et al., 2018).

#### Transport Model

In the monolayer model (Monolayer ABM in **Supplementary Materials**), concentrations depend only on depth, leading to a simple 1D model for diffusive transport in the medium. Mass flux at the medium-cell interface is a function of the *trans*-membrane concentration difference. The intracellular reaction equations are combined with the discretized 1D diffusion equation to give a system of ODEs.

In the spheroid model (Transport model in **Supplementary Materials**), oxygen and drugs are transported by diffusion from the medium and into the interior of the spheroid through the extracellular space in parallel with their uptake into cells. The combination of diffusion and intracellular metabolism results in a system of reaction-diffusion equations, expressed using the Method of Lines as a set of ordinary differential equations (ODEs), solved using a Runge–Kutta method. A lattice site may be vacant, in which case the whole site volume is extracellular and there is a single ODE for the concentration *C*, accounting for diffusion (and drug instability if relevant). In each site containing a cell there is an additional ODE for the intracellular compartment, accounting for exchange across the cell membrane and intracellular metabolism. In the case of oxygen, the molar rate of metabolism *M^′^* is modeled using Michaelis–Menten kinetics.

#### Pharmacokinetic/Pharmacodynamic (PKPD) Models

Hypoxia-activated prodrugs undergo a sequence of reactions in a cell; the model identifies up to two serial metabolites: Prodrug → Metabolite 1 → Metabolite 2 → untracked products, assuming stoichiometric conversion, where rates of metabolism (*M*′) are described by oxygen-dependent first order rate constants for metabolic consumption (*K_met_*) (detailed under Oxygen Dependence of Prodrug Metabolism in **Supplementary Materials**):

$$M^{\prime }=K_{met}C$$

The PKPD models assume that the intrinsic sensitivities of activators and targets to the cytotoxic metabolites are identical (Pharmacokinetic/Pharmacodynamic Models in **Supplementary Materials**). For SN30000 we further assume that Metabolite 1 is a cytotoxic, short-lived free radical (Anderson et al., 2014) that does not diffuse from the cell, while its downstream 1-oxide and nor-oxide metabolites are non-cytotoxic as demonstrated previously (Hicks et al., 2010; Wang et al., 2014; Gu et al., 2017). The previously validated PKPD model (Hicks et al., 2006, 2010), describes the probability of clonogenic cell killing (*P_kill_*) by SN30000 in a small time interval Δ*t* as the product of the killing rate constant *K_c_* and its rate of bioreductive metabolism *M*′ (to which the rate of generation of the transient intracellular cytotoxic radical is assumed to be proportional):

$$P_{kill}=K_{c}M^{\prime }\Delta t$$

The PR104A model assumes that Metabolites 1 and 2 are DNA interstrand crosslinking cytotoxins with sufficient stability to diffuse from the cell of origin. These metabolites have lifetimes defined by the first order intracellular rate constants for their subsequent reactions and instability in the medium; the PKPD model is consistent with the reported relationship between PR104A exposure (*C*) and clonogenic cell killing for single cells in Foehrenbacher et al. (2013a):

$$P_{kill}=K_{c}C\Delta t$$

When a cell is simultaneously exposed to two cytotoxic metabolites, the probability of survival is given by the product of the two survival probabilities:

$$P_{kill}=1-\left(1-P_{kill\,\;1}\,\right)(1-P_{kill\,\;2}\,)$$

### Statistical Tests

One-way ANOVA with the Holm–Sidak *post hoc* test was performed using SigmaPlot (Systat Software, v13.0). N.S, not significant (*P* > 0.05); ^∗^*P* < 0.05; ^∗∗^*P* < 0.01; ^∗∗∗^*P* < 0.001.

## Results

### Generation and Characterization of Activator and Target Cell Lines

To generate cells with high prodrug activation competence (activators), and cells that cannot activate prodrugs efficiently (targets), isogenic HCT116 cell lines were constructed as described in **Supplementary Figures 1A,B**. Briefly, *POR* was either knocked out (Su et al., 2013a) or an *N*-terminal truncated variant of POR (Foehrenbacher et al., 2013a) was expressed to modify net one-electron reductase activity under anoxia. Cells were also transfected with vectors for red or green fluorescent proteins to facilitate their detection in mixed cultures, and antibiotic resistance genes for clonogenic assay of cells in selective media.

**FIGURE 1:**
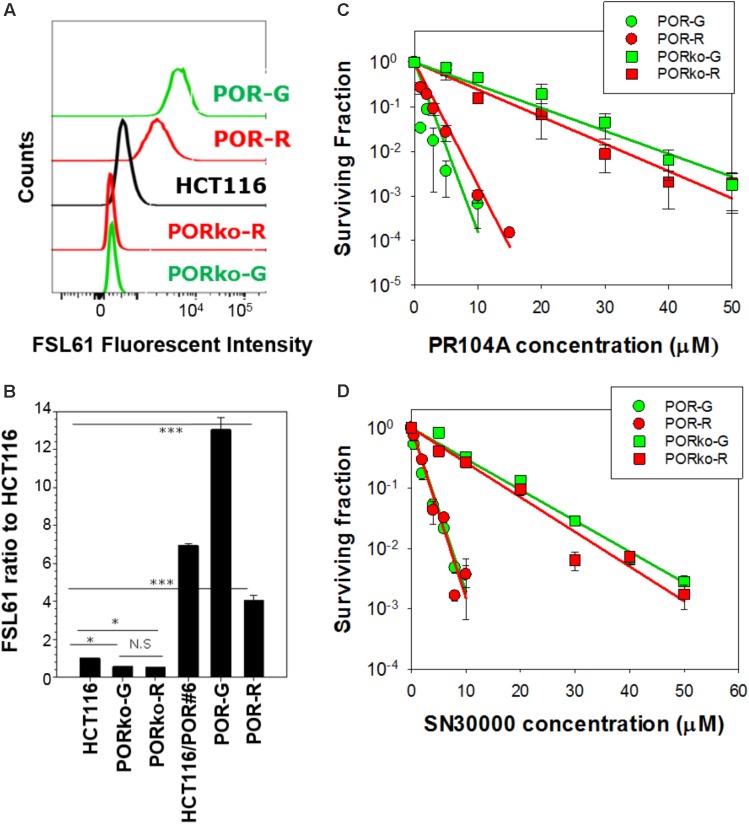
Characterization of high POR “activator” and POR-null “target” cell lines. **(A)** Flow cytometry profiles following incubation of cells with the fluorogenic one-electron reductase probe FSL61 under anoxia. **(B)** The median fluorescent intensity of FSL61 normalized to that in parental HCT116 cells. Values are means and SE of four cultures from two independent experiments. **(C,D)** Clonogenic survival of activators and targets after exposure of monolayer cultures (10^5^ cells/0.15 mL) to PR104A **(C)** or SN30000 **(D)** under anoxia for 1 h. Values are means and SE from three experiments (PR104A) or three biological replicates (SN30000). Lines represent linear regression fits.

P450 oxidoreductase-transfected activator clones labeled with mRuby (POR-R) or copGFP (POR-G) markers showed marked increases in one-electron reduction activity, assessed with the FSL61 fluorogenic probe (**Figure 1A**). POR-null target cell lines expressing TagRFP657 (PORko-R) or EGFP (PORko-G) fluorescent proteins, showed modest reductions in FSL61 metabolism. These changes relative to parental cells were statistically significant (**Figure 1B**). There was a greater differential (24-fold) for the POR-G/PORko-R pair than for the POR-R/PORko-G pair (eightfold). Clonogenic assays of monolayer cultures exposed to HAPs under anoxia demonstrated marked increases in the sensitivity of both activator cell lines to PR104A (**Figure 1C**) or SN30000 (**Figure 1D**).

### Development of Co-culture Spheroid Model

**FIGURE 2:**
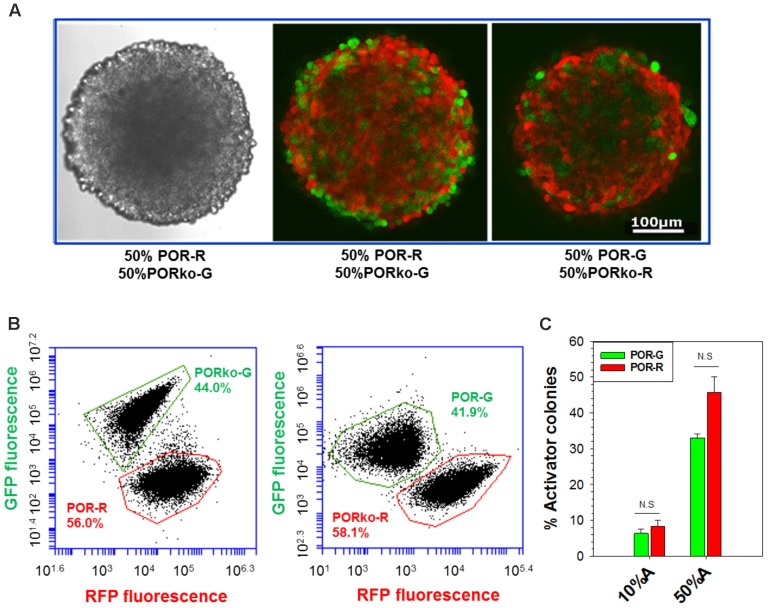
Analysis of activators and targets in spheroid co-cultures. Spheroids were grown for 4 days, without antibiotic selection, after seeding mixtures of activators and targets (3,000 cells/well). **(A)** Confocal images of spheroids generated from a 1:1 mixture of activator and target cells. Left: transmitted light. Center, right: fluorescence. **(B)** Flow cytometry analysis of fluorescent protein markers in cells from spheroids grown as in **(A)** then trypsinized. **(C)** The proportion of activators in spheroids grown from mixtures with 10% or 50% activators, determined by plating trypsinized spheroids in media with puromycin or G418 to identify activator and target clonogens, respectively. Mean and SE are from three to six independent experiments.

Spheroids were initiated in hanging-drop cultures, using differing proportions of activator and target cells, and grown for 4 days without antibiotic selection. Confocal microscopy demonstrated intimate mixtures of red and green fluorescent cells (**Figure 2A**) in proportions consistent with the seeding ratios (**Supplementary Figure 2**). Loss of image resolution was evident in central regions as expected given limited light penetration, a known shortcoming in confocal microscopy of spheroids (Pampaloni et al., 2013). We therefore employed flow cytometry to enumerate RFP- and GFP-tagged cells in trypsinized spheroids, demonstrating similar proportions to the 1:1 input ratio although with a possible reduction in POR-G cells (**Figure 2B**) consistent with the slower growth rate of POR-G cells in monolayer cultures (**Supplementary Table 1**). Similarly, fewer POR-G than POR-R cells were found in spheroid co-cultures when clonogens were quantified by plating in selective media, with puromycin to select for activators and G418 for targets (**Figure 2C**).

### Investigation of Bystander Effects in Spheroid Co-cultures

Prior to testing the cytotoxic activity of HAPs in mixed-lineage spheroids, we critically evaluated conditions for achieving uniform hypoxia in the spheroids by using a 2-nitroimidazole probe (Tercel and Pruijn, 2011) to label hypoxic cells with click chemistry. After transfer to an anaerobic chamber, the medium was changed six times using medium pre-equilibrated in the chamber (see the section “Materials and Methods”). Even under these conditions, much more uniform probe binding was achieved if the spheroids were also transferred to plates that had been similarly pre-equilibrated to remove oxygen, as demonstrated by both flow cytometry and confocal microscopy (**Supplementary Figure 3**). This reflects the high solubility and slow release of oxygen in polystyrene plasticware (Chapman et al., 1970).

**FIGURE 3:**
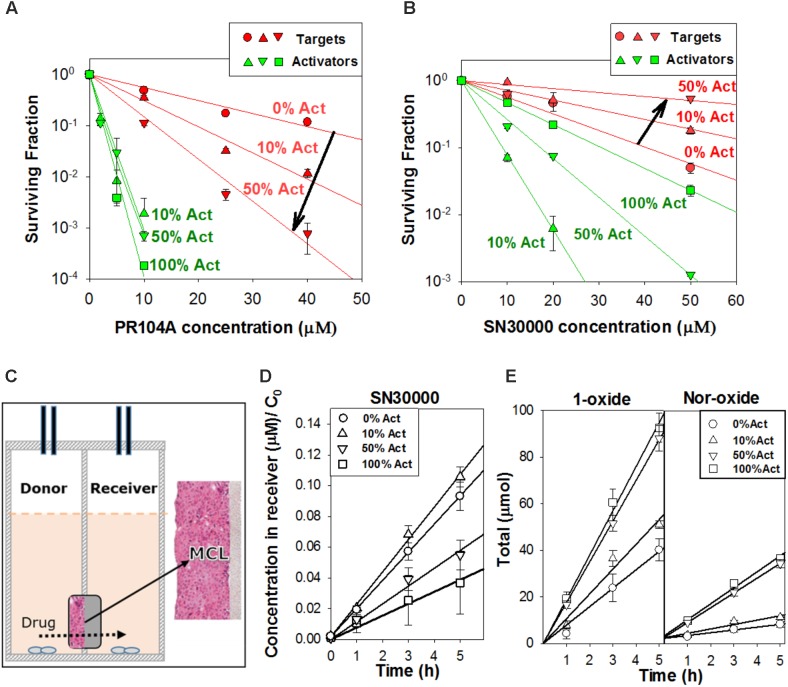
Investigation of bystander effects in co-culture spheroids grown from mixtures comprising 0, 10, 50, or 100% activators (Act). **(A)** Clonogenic survival of targets (PORko-R) and activators (POR-G) in co-culture spheroids after exposure to PR104A for 1 h under anoxia. **(B)** Clonogenic survival of targets (PORko-G) and activators (POR-R) in co-culture spheroids following SN30000 treatment for 1 h under anoxia. Lines are linear regression fits. **(C)** Apparatus for measuring diffusion of drug and metabolites through multicellular layers (MCL) in which layers of cells grow on a porous support membrane. Drug is added to the donor compartment and concentrations of drug and metabolites in the donor and receiver compartments are measured at intervals. **(D,E)** Diffusion of SN30000 and its metabolites through anoxic MCLs with different proportions of POR-R activators (Act) and PORko-G targets. **(D)** SN30000 concentrations in the receiver compartment, normalized to the initial SN30000 concentration in the donor (*C*_0_). **(E)** Total amount of metabolites (μmol) in the donor and receiver compartments. Lines represent linear regression fits. Means and SE are from six MCLs from three independent experiments.

We used this wash-and-transfer protocol to evaluate clonogenic cell killing of activators and targets, by HAPs, in anoxic spheroids (**Figures 3A,B**). Surviving clonogens were quantified by plating in selective media as above. When spheroids were exposed to PR104A, killing of PORko-R target cells markedly increased with increasing proportion of POR-G activator cells, suggesting that the active metabolites can diffuse out of activators and elicit killing of targets (**Figure 3A**). The same trend was also observed in smaller POR-G/PORko-R spheroids (**Supplementary Figure 4A**) and in spheroids grown from the POR-R/PORko-G activator/target pair (**Supplementary Figure 4B**), although PR104A potency was lower as expected given the lower one-electron reductase activity of POR-R than POR-G activators (**Figure 1B**).

In marked contrast to PR104A, increasing proportions of POR-R activators *reduced* killing of PORko-G targets after SN30000 treatment (**Figure 3B** and **Supplementary Figure 4C**). Given that the same trend was observed for killing of activators, we hypothesized that rapid metabolic consumption of SN30000 by activators compromised penetration of SN30000 into spheroids. This was confirmed by quantifying net diffusion of SN30000 through anoxic MCLs (**Figure 3C**) which was lowered by increasing proportions of activator cells (**Figure 3D**). Concentrations of the non-cytotoxic 1-oxide and nor-oxide metabolites (**Figure 3E**), which are downstream of the initial cytotoxic free radical metabolite (Hicks et al., 2010), increased with the proportion of activators, supporting this interpretation.

### Development of Agent-Based Models for HAP PKPD

**FIGURE 4:**
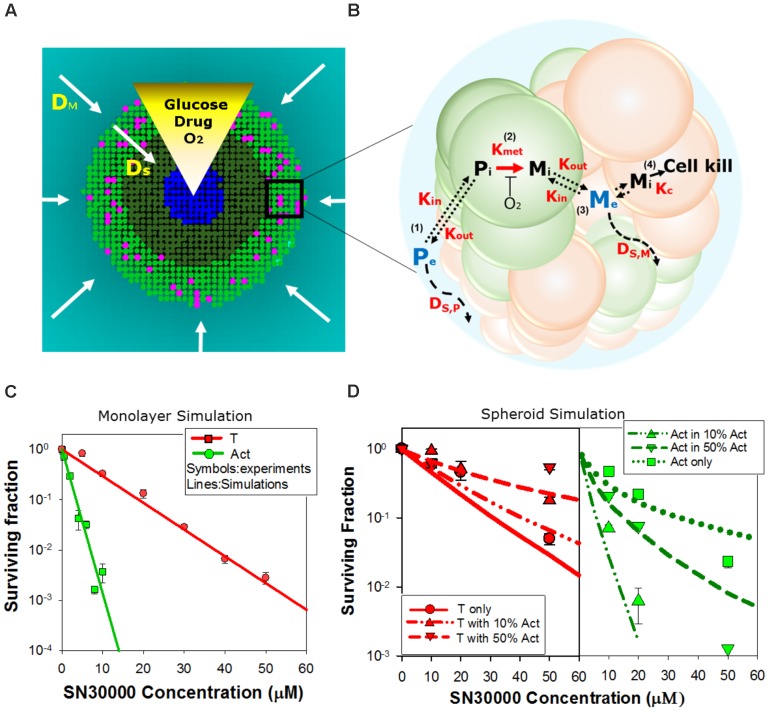
Agent-based model (ABM) of SN30000 diffusion and cytotoxicity in spheroids. **(A)** The ABM simulates diffusion gradients of oxygen, glucose, drugs and their metabolites in the 3D spheroid and surrounding medium (arrows). In the model, illustrated here as a transverse section through the midplane of a 4-day-old spheroid, these gradients control proliferation (mitotic cells, pink), fraction of hypoxia (O_2_ < 4 μM, dark green), central necrosis (blue) and cellular response to external stimuli including drug treatment. Diffusion in the unstirred bulk medium and the spheroid are described by diffusion coefficients D_M_ and D_S_, respectively. If severe hypoxia (O_2_ < 0.15 μM) persists for more than 24 h, cells are ‘tagged’ for death (blue) and will become necrotic. **(B)** Schematic diagram illustrating the cellular pharmacokinetic/pharmacodynamic (PKPD) model: (1) extracellular prodrug (*P*_e_) exchanges with intracellular prodrug (P_i_) with transfer across the cell membrane determined by the rate constants, *K*_in_ and *K_out_*; (2) *P*_i_ is metabolized to intracellular cytotoxic metabolite (*M*_i_) in activators (green) with first order rate constant *K_met_*; (3) *M*_i_ may efflux from cells to initiate bystander killing via uptake by surrounding target cells (red); (4) cell kill probability is a compound-specific function of exposure to *M*_i_ and potency of *M*_i_ (*K*_c_). **(C,D)** Model predictions of clonogenic survival of activators (Act; POR-R) and targets (T; PORko-G) in monolayer cultures **(C)** or spheroids **(D)** after SN30000 treatment under anoxia. Lines are simulation outputs and symbols are measured values (redrawn from **Figures 1D**, **3B**, respectively).

We next asked whether activator and target cell killing in spheroids can be predicted from their sensitivity to HAPs in monolayer cultures, taking into account the pharmacokinetics of the prodrugs and their metabolites within the spheroids. To do this we utilized ABMs that describe the growth and drug response of each individual cell in the 3D spheroid, in the context of the time-dependent concentration gradients imposed by the diffusion and reaction of oxygen, glucose (controlling growth) and prodrugs and their metabolites during HAP exposure under anoxia (**Figure 4A** and **Supplementary Materials**) (Mao et al., 2018). In this model, diffusion occurs through the unstirred bulk medium and extracellular spaces in the spheroid, metabolism of O_2_, glucose, and prodrugs occurs intracellularly, and prodrugs and their metabolites exchange across the plasma membrane (**Figure 4B**). The diffusion and cytotoxicity of HAP metabolites is explicit in the model, enabling critical evaluation of the magnitude of bystander effects. Spheroid growth was modeled as described, using doubling times of 38 h for POR-G and 31 h for the other cell lines (ca. 30% longer than log-phase monolayers, **Supplementary Table 1**) which gave spheroid diameters on day 4 consistent with observation and activator/target ratios consistent with **Figures 2B,C**. PKPD model parameters, and data sources, are provided in **Supplementary Tables S2** (SN30000), **S3** (PR104A), and **S4** (O_2_), along with any adjustments of previously reported parameter values required to optimize model fits.

### Simulation of SN30000 Activity by a ‘No Bystander Effect’ Model

For SN30000 simulations, a ‘no bystander effect’ model was used in which the rate of cell killing is proportional to the rate of SN30000 metabolism to its DNA-reactive oxidizing free radical metabolite, under the assumption that the latter is too reactive to escape the activator cells. To constrain the model, we first used monolayer ABM (where all cells are exposed to the same concentrations of O_2_ and drug) to simulate clonogenic killing by SN30000 of activators and targets in anoxic monolayers (**Figure 4C**) using PKPD parameters for HCT116 cells (**Supplementary Table 2**). To optimize the fits (**Figure 4C**) the measured rate constant for SN30000 metabolism (*K*_*met*, 0_) in parental HCT116 cells (1.88 min^-1^) was adjusted to 1.3 min^-1^ for PORko-G targets and 14 min^-1^ for POR-R activators consistent with the measured ca. 10-fold differential in FSL61 metabolism (**Figure 1B**). To simulate killing in spheroids, the parameters estimated for monolayers were used in the spheroid ABM, together with measured estimates of the diffusion coefficient of SN30000 in the medium and MCLs. The model accurately predicted sensitivity of activators and targets to SN30000 in spheroids (**Figure 4D**), with a strong correlation between predicted and measured log cell kill over the whole dataset (*R*^2^ = 0.838). This indicates that the penetration limitation imposed by rapid metabolic consumption of SN30000 (**Figure 3D**) quantitatively accounts for the apparent resistance of cells in spheroids relative to monolayers.

### Simulation of PR104A Activity: The ‘PR104H+M’ Model

**FIGURE 5:**
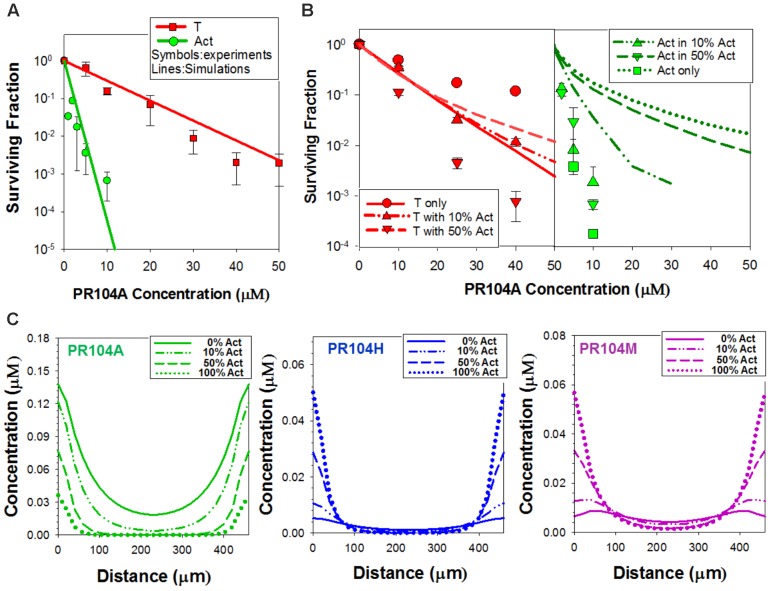
PR104A simulation using the ‘PR104H+M’ agent-based model. **(A,B)** Model predictions of clonogenic survival of activators (Act) and targets (T) after exposure to PR104A for 1 h under anoxia in monolayer cultures **(A)** or in co-culture spheroids **(B)** using experimentally determined parameters for transport and reactivity of PR104A and its active metabolites PR104H and PR104M (detailed in **Supplementary Table 3**). Symbols are measured values (redrawn from **Figures 1C**, **3A**) and lines represent simulation results from monolayer ABM **(A)** and spheroid ABM **(B)**. **(C)** Intracellular concentrations of PR104A, PR104H, and PR104M predicted by the PR104H+M model across the central transverse section of a representative co-culture spheroid following 1 h exposure to 30 μM PR104A under anoxia.

We took a similar approach to simulate PKPD of PR104A in mixed spheroids, again constraining the model by fitting the dose-response curves for cell killing in anoxic monolayers. Our first bystander effect ABM for PR104A assumed that its DNA interstrand-crosslinking metabolites, PR104H and PR104M, diffuse from the cell of origin and elicit bystander killing. The parameter set previously reported for HCT116 and HCT116/POR#6 (Foehrenbacher et al., 2013a) (**Supplementary Table 3**) provided excellent agreement with the monolayer survival data (**Figure 5A**). However, using the measured transport parameters for PR104A and its metabolites (Foehrenbacher et al., 2013a), the model predicted that high proportions of activators would compromise killing of both activators and targets (as for SN30000), which was opposite to the observed effect (**Figure 5B**). Simulated concentration gradients of intracellular PR104A, PR104H, and PR104M across the transverse section of the center of the spheroid identified that the diffusion distance of PR104H and PR104M is too limited to compensate for metabolic consumption of PR104A in spheroids at high activator ratios (**Figure 5C**). The conspicuous mismatch between model and observation led us to critically re-evaluate the cellular pharmacology of PR104A.

### Cellular Pharmacology of PR104A

**FIGURE 6:**
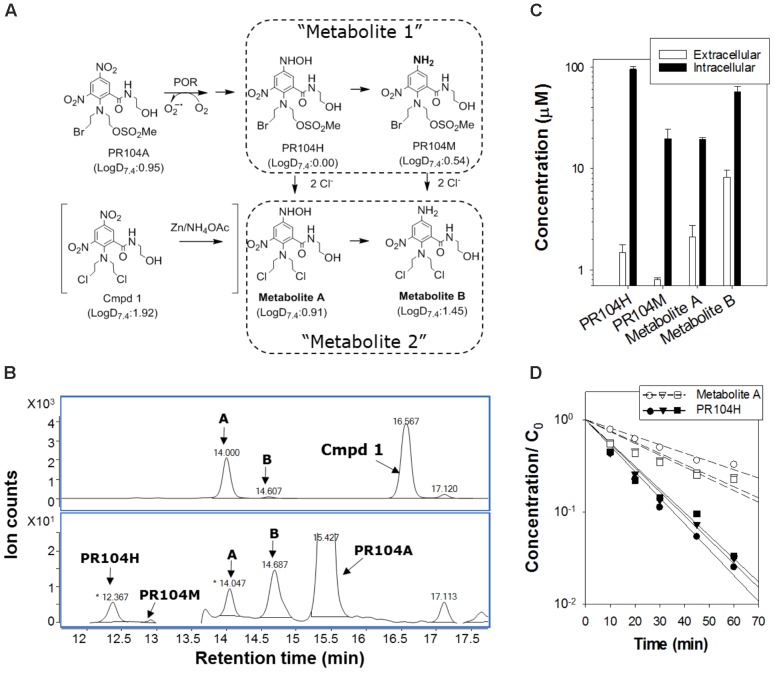
Identification of extracellular activated nitrogen mustard metabolites from PR104A. **(A)** Metabolic activation pathway of PR104A under anoxia. Metabolites A and B result from displacement of the mesylate and bromo leaving groups of the initial activated metabolites PR104H and PR104M, respectively. Metabolite A was also synthesized from the dichloro analog of PR104A (shown in brackets). LogD at pH 7.4 (LogD_7.4_) was estimated as a measure of lipophilicity using trained ACD software as detailed in **Supplementary Figure 7**. **(B)** Representative HPLC chromatograms of reduced metabolites following zinc dust reduction of the dichloro analog of PR104A (upper panel) and in extracellular medium from POR-R cultures incubated with PR104A (100 μM for 3 h) under anoxia (lower panel). **(C)** Intracellular and extracellular concentrations of metabolites in POR-R cultures after 100 μM PR104A for 3 h. Values are means and range for two independent experiments. **(D)** Stability of PR104H and Metabolite A in culture medium at 37°C by LC-MS/MS with stable isotope internal standards as detailed in **Supplementary Figure 6**. Concentrations are normalized to initial PR104A concentrations, *C*_0_ (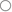: 10 μM, 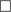: 30 μM, △: 100 μM PR104A).

Surveying metabolites of PR104A in the extracellular medium of anoxic POR-R monolayers by mass spectrometry identified derivatives of PR104H and PR104M with masses consistent with chloride ion displacement of the mustard leaving groups and with isotope patterns identical to the corresponding theoretical spectra (**Supplementary Figure 5**). The inferred dichloro derivatives (Metabolites A and B; **Figure 6A**) were confirmed by zinc dust reduction of the corresponding dinitro compound (**Figure 6B**). Quantifying the major intracellular and extracellular metabolites 3 h after addition of PR104A showed that PR104H was the major intracellular metabolite but extracellular concentrations of Metabolite B were fivefold higher than for PR104H (*P* = 0.02; **Figure 6C**). A similar profile of extracellular metabolites was observed in anoxic SiHa cultures (**Supplementary Figure 6**). Octanol:water partition coefficients at pH 7.4 (logD_7.4_) for the metabolites, calculated using a training set of 153 compounds with measured values including 37 dinitrobenzamide mustards related to PR104A (**Supplementary Figure 7** and **Supplementary Table 5**), showed that Metabolite A and B are more lipophilic than PR104H or PR104M (**Figure 6A**). Metabolite B was not synthetically accessible, but we purified Metabolite A from the zinc dust reduction mixture and studied its stability in culture medium; this demonstrated a fourfold longer half-life than PR104H (**Figure 6D**), reflecting the known lower reactivity of Cl than Br or mesylate leaving groups in nitrogen mustards (Atwell et al., 2007). A similar increase in stability can be expected for Metabolite B. These physicochemical properties (higher lipophilicity and greater stability) are consistent with the observed accumulation of Metabolite B in extracellular medium, and suggested it as a candidate mediator of the PR104A bystander effect.

### Simulation of PR104A Activity: The ‘Dichloro Metabolites’ Model

**FIGURE 7:**
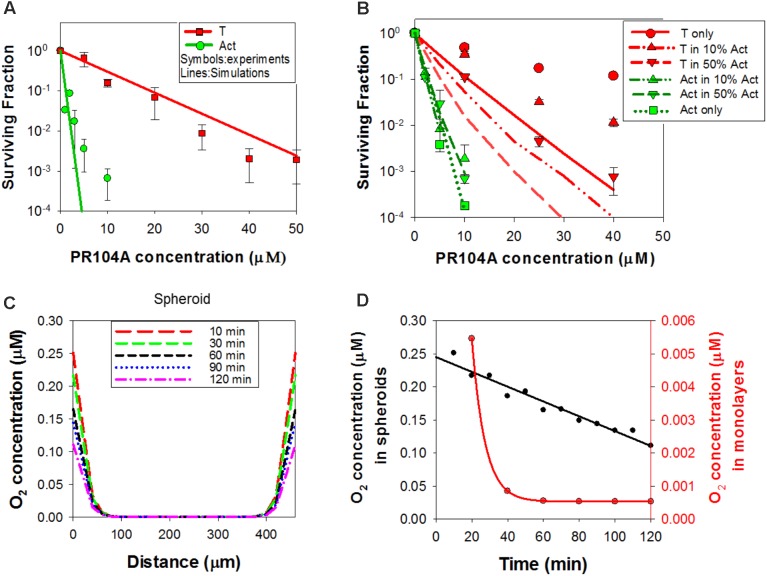
Simulation of PR104A activity in monolayers and spheroids by the ‘dichloro metabolites’ model under anoxia. **(A)** Estimated clonogenic survival of activators and targets in anoxic monolayers exposed to PR104A. **(B)** Predicted clonogenic survival of targets and activators in anoxic co-culture spheroids. **(A,B)** Symbols are experimental values (re-drawn from **Figures 1C**, **3A**, respectively) and lines are simulation outputs. **(C)** Simulated time-dependent intracellular concentration of O_2_ across the center of a 4-day HCT116 spheroid (diameter 426 μm) in medium containing an initial concentration of 2.5 μM O_2_, with zero O_2_ in the gas phase. The medium is well-mixed at time zero (simulating addition of drug solution containing O_2_), and unmixed thereafter. **(D)** Simulated intracellular O_2_ concentration at the surface of the spheroid (black) and in HCT116 monolayers (red; 6.7 × 10^6^ cells/mL, with 0.15 mL medium per well) over time, with addition of bolus O_2_ to an initial concentration of 2.5 μM as for **(C)**.

In order to test whether the dichloro metabolites can account for the observed bystander effects of PR104A in spheroids, we developed a new ‘dichloro metabolites’ model in which the proximal cytotoxin (Metabolite 1) represents PR104H and PR104M (using parameters representative of these moderately reactive mustards) and the distal cytotoxin (Metabolite 2) represents the more stable Metabolites A and B (**Figure 6A**). Optimized parameters are detailed in **Supplementary Table 3**. Briefly, intracellular and extracellular pharmacokinetics of PR104A metabolites in anoxic HCT116/POR#6 single cell suspensions were modeled by optimizing the reaction parameters for the metabolites, with *K*_*met*, 0_ for metabolism of PR104A unchanged from the published value (Foehrenbacher et al., 2013a) (**Supplementary Figure 8**). This provided a parameter set for the notional metabolites 1 and 2 that was consistent with the physicochemical properties of the Br/mesylate and dichloro metabolites, respectively (**Supplementary Table 3**). Using cell killing in monolayers, *K*_*met*, 0_ for POR-G and PORko-R were fitted, adjusting measured *K*_*met*, 0_ in HCT116 and HCT116/POR#6 (Foehrenbacher et al., 2013a) based on the FSL61 data in **Figure 1B**. Fitting the potency parameter *K_c_* for metabolite 2 gave an estimate of 0.05 mM^-1^s^-1^ (0.092 mM^-1^s^-1^ for PR104H). This is broadly consistent with the lower cytotoxic potency of synthetic Metabolite A than PR104H in oxic cell cultures (**Supplementary Figure 9**). The resulting model gave good agreement with the experimental cell kill values for monolayers (**Figure 7A**). Using these same parameters in the spheroid ABM now correctly predicted an increase in cell killing as the proportion of activators is increased, although the model overestimated potency against targets by twofold to threefold (**Figure 7B**).

**FIGURE 8:**
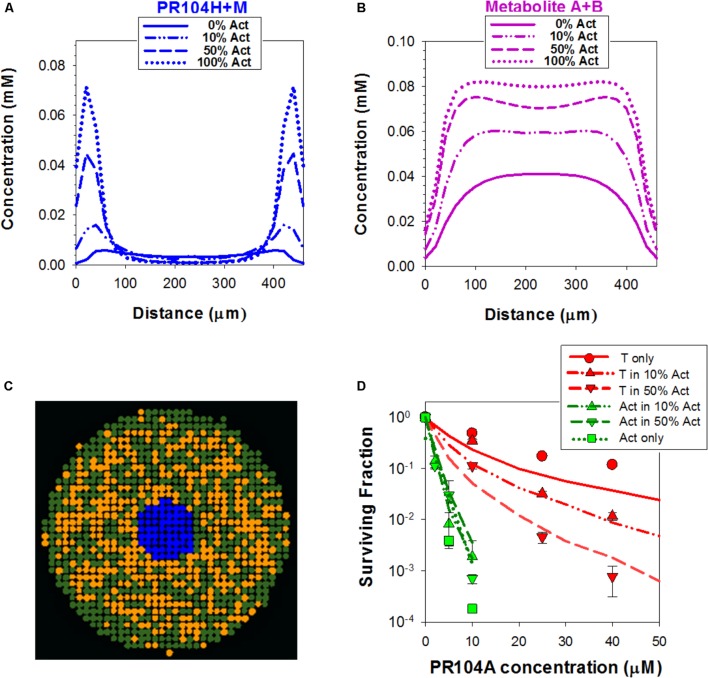
Simulation of PR104A activity by the ‘dichloro metabolites’ model in spheroids. **(A,B)** Estimated intracellular concentrations of PR104H+M **(A)** and Metabolites A+B **(B)** in co-culture spheroids after 1 h exposure to 30 μM PR104A. **(C)** Representative central transverse section of a simulated 3D spheroid (comprising PORko-R target cells only, to minimize PR-104A concentration gradients) illustrating the spatial distribution of cells tagged for death by active metabolites (orange) at the end of the exposure. **(D)** Model estimations of clonogenic survival of activators and targets in co-culture spheroids treated with PR104A. Lines are simulation outputs and symbols are measured values redrawn from **Figure 3A**.

This discrepancy led us to consider the effect of oxygen dissolved in the PR104A stock solutions used to initiate the “anoxic” drug exposures; given the high solubility of oxygen in methanol (2.16 mM at 20°C) (Tokunaga, 1975), the initial average oxygen concentration in the spheroid cultures immediately after adding PR104A was 2.5 μM, leading to a model-based prediction of an initial oxygen concentration of 0.25 μM at the spheroid surface (**Figures 7C,D**). In the monolayer experiments, simulated oxygen contamination was much less severe (**Figure 7D**) as oxygen contamination is removed more rapidly by cellular consumption at the higher cell/medium ratios. Given the exquisite sensitivity of PR104A metabolism to oxygen (*K*_*O*2_ = 0.126 μM) (Hicks et al., 2007), this contamination led to a ABM prediction of appreciably suppressed metabolite formation near the surface of the spheroid. Coupled with diffusion of metabolites into the bulk medium, this led to steep metabolite concentration gradients at the surface, while the dichloro metabolites were predicted to diffuse efficiently into the center of the spheroid (**Figures 8A,B**). The resulting spatial pattern of cell killing, sparing cells at the surface, is illustrated in **Figure 8C**. Notably, the final spheroid model incorporating the two classes of metabolites and the effects of oxygen contamination provided a good description of the experimental data (**Figure 8D**), with a correlation coefficient of 0.868 for model prediction versus measurement.

**FIGURE 9:**
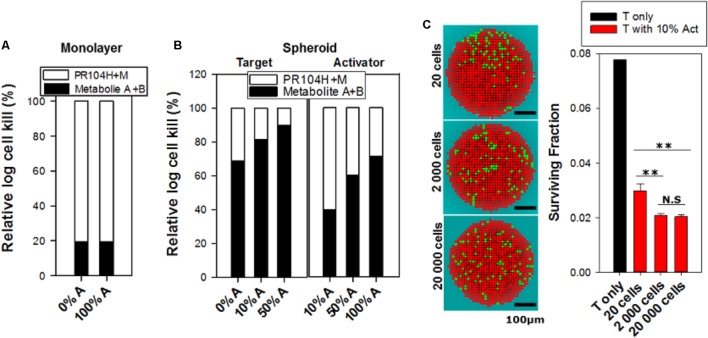
Relative contribution of dichloro metabolites (Metabolites A+B) to killing of cells in monolayers and spheroids, and predicted cell killing in spheroids with varying spatial heterogeneity of prodrug activation, following anoxic exposure of PR104A for 1 h. **(A)** Relative log kill of activators or targets by PR104H+M or Metabolites A+B in monolayer cultures treated at PR-104A concentration giving 90% kill (17 μM for targets and 1 μM for activators). **(B)** Contribution of PR104H+M and Metabolites A+B to killing of targets and activators in spheroid co-cultures. Relative log cell kill by either metabolite was estimated for 20 μM PR104A for targets and 5 μM for activators, which are the concentrations giving 90% kill in spheroids comprising 100% targets and 100% activators, respectively. **(C)** Images of spatial distribution of activators (green) and targets (red) in transverse sections through the center of the 3D spheroids, simulated by the ABM using different initial cell numbers (20, 2,000, and 20,000 cells) and grown until they reached the same diameter (∼430 μm). The same doubling time (16 h) was used for activators and targets to give the same proportion of activators (10%) in each case. Cell death due to severe hypoxia was not included in the ABM growth model in order to avoid any random effects of difference in necrotic fraction. The right graph shows the effect of spatial heterogeneity of POR expression on bystander killing of targets after treatment with 40 μM PR104A for 1 h. Values are means and SE for six simulations with different initial random distributions of activators.

We used this final model to dissect the contributions of PR104H+M and the dichloro metabolites, demonstrating that cytotoxicity in activators and targets is largely dependent on PR104H+M in monolayer cultures (**Figure 9A**). In contrast, in spheroids, the PR104H+M contribute to killing of activators but have little activity against targets while the dichloro metabolites play the major role in target cell killing. These relative contributions are summarized in **Figure 9B**, and emphasize that the dichloro metabolites are the main bystander mediators.

We also used this final model to simulate the effects of differing spatial distributions of activators on the efficiency of bystander effects by initiating spheroid growth *in silico* using fewer cells (at a constant activator/target ratio of 10%) but growing each to the same size. Seeding with just 20 cells in total led to highly variable and non-uniform distributions compared to spheroids initiated with 2,000 or 20,000 cells and significantly reduced the model-predicted efficiency of killing of target cells (**Figure 9C**). This demonstrated the role of spatial heterogeneity of the metabolically competent activators in killing of targets, although it is notable that the analysis demonstrated that PR104A activation results in marked bystander effect killing over distances of many cell diameters.

## Discussion

In this study, we report an integrated experimental and mathematical modeling approach for addressing the substantial challenge of characterizing the PKPD of anticancer agents in tumors at a cellular level of resolution. Using 3D cell cultures in conjunction with ABM makes it possible to investigate spatial scales of tens to hundreds of microns that are relevant to extravascular regions in tumor tissue. Our models include neither the vascular networks that define these regions, nor the other stromal elements that contribute to tumor microenvironments, but 3D spheroid and multilayer tumor cell cultures are now well-established as experimental models suitable for investigating drug, oxygen and nutrient gradients in tumor tissue (Hirschhaeuser et al., 2010; Schutze et al., 2015; Baek et al., 2016; Dovmark et al., 2017; Weltin et al., 2017). These tools are particularly valuable for investigating bystander effects that potentially decouple targeting of prodrugs, antibody drug conjugates and nanoparticles from their pharmacodynamic effects.

In the present approach, we generate microregional heterogeneity of HAP activation by spatially varying reductase expression, rather than varying hypoxia, in 3D tissue models; reductase expression is more amenable to experimental manipulation and measurement than oxygen gradients. We and others have previously used this general approach to demonstrate bystander effects from prodrugs activated by oxygen-insensitive bacterial reductases in MCLs (Wilson et al., 2002; Meng et al., 2012; Mowday et al., 2016), but metabolism kinetics and metabolite profiles from these often differ from endogenous one-electron reductases responsible for activation of HAPs under hypoxia (Helsby et al., 2004; Wilson et al., 2007). A previous MCL co-culture system with POR-overexpressing A549 cells provided suggestive evidence for a hypoxia-mediated bystander effect from dinitrobenzamide predecessors of PR104A but not for tirapazamine (Wilson et al., 2007). However, modeling tools were not available to dissect the competing effects of prodrug metabolism on prodrug penetration and formation/diffusion of bystander metabolites – which we show in the present study to be critically important.

The loss of activity of SN30000 against low-POR target cells when the proportion of activators increases (**Figure 3B**) was shown by model simulation to be entirely accounted for by the observed penetration impediment (**Figure 3D**), resulting in an excellent fit to measured killing (**Figure 4D**). The spheroid model was parameterized using the PKPD relationship for killing in monolayers plus the diffusion coefficient and kinetics of metabolic consumption of SN30000 measured in MCLs. This result demonstrates that any bystander effect of SN30000 must be trivially small. The converse pattern with PR104A (increasing target killing with increasing activators) clearly demonstrated the existence of a bystander effect (**Figure 3A**), but the ABM revealed important features of the pharmacology of this HAP. In particular, there is a significant penetration impediment that is not evident from the cell killing data, but is demonstrated by the model which predicts lower concentrations of PR104H+M in the center of spheroids with high activator ratios (**Figure 5C**). This impediment is less severe than for SN30000, due to slower kinetics of reduction (**Supplementary Tables 2**, **3**), but importantly the penetration problem is more than compensated by the efficient bystander effect of the downstream metabolites.

A second feature revealed by the spheroid model is that the well-studied PR104H and M metabolites cannot account for the observed bystander effect. We show that inclusion of the dichloro metabolites (formalized as “Metabolite 2” in the ABM; **Figure 6A**) substantially changes model predictions, and is essential for correspondence with experimental observations. Dichloro metabolites of PR104A have been noted previously, in anoxic SiHa cell cultures (Patterson et al., 2007) but their significance had not been appreciated. The ABM demonstrates that the dichloro metabolites are the main contributors to bystander effects; however, the two metabolite types are complementary. In fact the bromo/mesylate metabolites, PR104H+M, are more effective in killing cells in monolayers or near the spheroid surface (and by inference, perivascular cells in tumors) as they are less readily washed out into the medium (or blood) than the more lipophilic dichloro metabolites (**Figures 8A,B**, **9A,B**). Thus generating multiple active metabolites with different physicochemical properties is an effective strategy for extending cell killing across different microenvironmental niches in tumors. The considerable diffusion range of the dichloro metabolites is demonstrated by modeling bystander effects in spheroids with highly non-uniform distributions of high-POR activator cells, which showed only a minor reduction in target cell killing (**Figure 9C**). Given the spatial scales involved, this indicates that the bystander metabolites of PR104A have the ability to diffuse from hypoxic zones to elicit significant killing in adjoining oxic regions.

An advantage of the flexibility of the ABM approach is that it facilitates modeling the whole experimental system, not just the spheroid, incorporating for example concentration gradients in unstirred medium in the cultures and oxygen contamination when drugs are introduced. We show that the latter has a material effect on activity of PR104A and was a necessary addition to the ABM in order to predict activity of this highly oxygen sensitive HAP (Hicks et al., 2007) in spheroid co-cultures (**Figures 7**, **8D**).

## Conclusion

In conclusion, agent-based modeling of the diffusion, metabolism and cytotoxicity of HAPs in combination with experimental determination of model parameters in monolayer and 3D cell cultures has provided a comprehensive description of the ‘micropharmacokinetics’ of SN30000 and PR104A. The PR104A model demonstrates that bystander effects are important not only for overcoming spatial heterogeneity of reductase expression (or hypoxia) but also for overcoming prodrug penetration limitations imposed by rapid metabolic consumption. The latter is a key consideration with respect to the use of predictive biomarkers for clinical use. Specifically, an efficient bystander effect ensures a monotonic relationship between tumor cell killing and biomarkers that predict rates of HAP metabolism (reductase expression and hypoxia), over a wide range of rates, whereas in the absence of a bystander effect high rates of activation may suppress antitumor activity. More generally, the tools validated through the present study have broad utility in understanding the PKPD of anticancer agents that release an active drug in the tumor microenvironment and in supporting the development of improved prodrugs, antibody-drug conjugates and nanoparticle delivery systems.

## Author Contributions

CH, JW, FP, WW, and KH designed the experiments. GB developed the monolayer and spheroid ABM models. CH performed the experiments and ran the simulations. JW assisted with generation and characterization of activator and target cell lines (**Supplementary Figure 1** and **Figure 1**) and KP performed the experiments with SiHa cells (**Figure 6D** and **Supplementary Figures 6**, **9**). CH, GB, KH, and WW wrote the manuscript. FP, JW, and KP revised the manuscript.

## Conflict of Interest Statement

The authors declare that the research was conducted in the absence of any commercial or financial relationships that could be construed as a potential conflict of interest.
