# Supplementary material for: Bystander Effects of Hypoxia-Activated Prodrugs: Agent-Based Modeling Using Three Dimensional Cell Cultures
Source: Front Pharmacol. 2018 Sep 18;9:1013. doi: 10.3389/fphar.2018.01013 (PMC6153434; doi:10.3389/fphar.2018.01013)
Supplement: Supplementary file 1 [file Table_1.pdf]

SUPPLEMENTARY MATERIALS

Bystander Effects of Hypoxia-Activated Prodrugs: Agent-based Modelling Using Three Dimensional Cell Cultures

Cho Rong Hong[1], Gib Bogle[2,3], Jingli Wang[1], Kashyap Patel[1], Frederik B. Pruijn[1], William R. Wilson[1,3*] and Kevin O.Hicks[1,3]

[1]Auckland Cancer Society Research Centre, University of Auckland, Auckland, New Zealand.
[2] Bioengineering Institute, University of Auckland, Auckland, New Zealand.
[3] Maurice Wilkins Centre, University of Auckland, Auckland, New Zealand.

Corresponding Author
Professor William R. Wilson, Auckland Cancer Society Research Centre, University of Auckland, 85 Park Rd, Grafton, Auckland 1023, New Zealand. Ph: 64-99236883. Email: wr.wilson@auckland.ac.nz

TABLE OF CONTENTS

SUPPLEMENTARY METHODS 3
ABM Simulation 3
Spheroid ABM 3
Spheroid Growth Model 3
Transport Model 4
Oxygen Dependence of Prodrug Metabolism 5
Monolayer ABM 5
Pharmacokinetic/Pharmacodynamic (PKPD) Models 6
SUPPLEMENTARY FIGURES 8
SUPPLEMENTARY FIGURE 1 8
SUPPLEMENTARY FIGURE 2 10
SUPPLEMENTARY FIGURE 3 11
SUPPLEMENTARY FIGURE 4 12
SUPPLEMENTARY FIGURE 5 14
SUPPLEMENTARY FIGURE 6 15
SUPPLEMENTARY FIGURE 7 16
SUPPLEMENTARY FIGURE 8 17
SUPPLEMENTARY FIGURE 9 18
SUPPLEMENTARY TABLES 19

SUPPLEMENTARY TABLE 1. Population doubling time of parental HCT116, activator and target cell lines, determined in monolayer cultures. 19
SUPPLEMENTARY TABLE 2. SN30000 PKPD parameters 20
SUPPLEMENTARY TABLE 3. PR104A PKPD parameters 21
SUPPLEMENTARY TABLE 4. Oxygen parameters 23
SUPPLEMENTARY TABLE 5: Measured and calculated octanol:water partition coefficients at pH 7.4 ($LogD_{7.4}$) for a training set of nitrobenzamide mustards 24
REFERENCES 26

SUPPLEMENTARY METHODS

ABM Simulation

Monolayer ABM simulations were run on desktop Windows PCs (Intel core I7 processor) from a Qt (Qt Company, *https://www.qt.io/*) graphical user interface passing parameters to a DLL built with Fortran95. For parameter estimation grid searches were performed to minimize the sum of squared deviations from predicted survival probability or extracellular or intracellular concentrations. Alternatively in the case of the spheroid ABM the program was run on a high performance supercomputer cluster operated by the New Zealand eScience Infrastructure. For modelling the bystander effect, two different cell lines (activators and targets) are simulated, each with their own rates of growth and ability to metabolize prodrug to active metabolites. Spheroid growth for 4-5 days was simulated after randomly assigning the initial seeding number of cells to lattice positions to achieve the appropriate cell number and necrotic core diameter at treatment size. Intracellular drug concentrations and cell survival probabilities were calculated from the PKPD model for each cell and compared to experimental values.

Spheroid ABM

The on-lattice agent-based spheroid model is described fully elsewhere (Mao et al., 2018). Each cell in the spheroid is simulated as an autonomous agent with its individual history, phenotype and fate in response to drug exposure. The model can simulate oxygen, glucose and drugs (with up to two metabolites). In the experiments reported here the medium was kept well-supplied with glucose, and as a result only oxygen affected cell growth and survival. As a consequence glucose was turned off in the simulations, and therefore it has been omitted in the following summary of the model.

Spheroid Growth Model

The spheroid grows in a specified volume and depth of unstirred medium, containing dissolved oxygen; the whole culture is modelled, rather than the spheroid in isolation. The geometry of the model is lattice-based, cells occupying cubic sites on a regular 3D lattice. The lattice spacing is determined from the estimated number of cells/mm^3, by equating this to the number of sites/mm^3. Based on an estimate of 500,000 HCT116 cells/mm^3, the volume of a cubic lattice site is 2.0 pL (giving a lattice spacing of 12.6 µm), and the cell volume fraction of 0.5 previously determined in MCLs (Foehrenbacher et al., 2013) implies an average cell volume of V_{ave} = 1.0 pL. A nominal cell volume V_n is defined as $V_{ave}/1.2$. Cells grow in volume at a rate depending on the local oxygen concentration and divide when volume reaches a randomly-set value V_{div} in the range (1.6 ± 0.15)*V_n. (This formulation ensures that V_{ave} is midway between $V_{div}/2$ and V_{div}). The maximal growth rate is determined from the population doubling time (T_d) estimated in spheroids and cell growth rate decreases in proportion to the oxygen consumption rate. When a cell divides the daughter cell occupies an adjacent empty lattice position; if a vacancy does not exist cells are moved radially outwards to create one. As the spheroid grows the gradient of oxygen concentration within both the medium and spheroid increases (See Fig. 4A), as discussed below, and the ambient oxygen level in the spheroid interior falls. Cells below a critical oxygen concentration (<0.15 µM) for 24 h are tagged to die and undergo cytolysis after a further 24 h, leading to development of central necrosis matching that observed in HCT116 spheroids (Mao et al., 2018). Co-cultures are simulated using this model with appropriate seeding numbers of activator and target cells, each

with its characteristic doubling time. Spheroids grow to form a heterogeneous distribution of activators and targets from an initial random mixture (illustrated in Figure 9C).

Transport Model

The model employs two 3D grids and two solvers, one grid for the whole volume of medium, and an embedded much finer grid occupying a very small cubic region at the bottom of the well - the lattice discussed above - within which the spheroid growth is simulated. The solution on the coarse grid provides the boundary conditions for the solution within the lattice, and aggregated cell fluxes of constituents become sinks/sources within the coarser grid. This two-solver approach ensures that the changing fields of concentration within the medium are correctly accounted for. This is particularly important for accurate simulation of oxygen. As the spheroid grows and oxygen consumption increases, the concentration gradient within the medium steepens and the concentration at the spheroid boundary is steadily depressed. This decrease in boundary concentration has a significant influence on spheroid growth. Although drug dosing duration is typically too short to cause much change in drug levels in the medium, the rate at which drug metabolites escaping from the spheroid are transported away into the medium can have a significant effect on metabolite levels in the spheroid, again showing the importance of realistic simulation of medium processes. The way the two solvers function together is described in detail in (Mao et al., 2018) and will not be reiterated here. In the following attention is restricted to the solution for intra- and extracellular concentrations within the lattice.

Boundary concentrations are computed in each time step on the boundary of the cube occupied by the lattice. Oxygen and drugs are transported by diffusion from the boundary and into the interior of the spheroid through the intercellular space in parallel with their uptake into cells. The combination of diffusion and intracellular metabolism results in a system of reaction-diffusion equations, expressed as a set of ordinary differential equations (ODEs) using the Method of Lines. A parallelized Runge-Kutta-Chebychev algorithm is employed to solve these equations, updating intra- and extracellular concentrations of the constituents each time step. The solver accounts for the possibility that a lattice site (a cube of volume Δx^3) may be unoccupied by a cell, in which case the whole site volume is extracellular. In each site containing a cell, there is exchange across the cell membrane. In the case of a vacant site there is simply one ODE for the concentration C at site (i,j,k):

$$\frac{dC_{ijk}}{dt} = \left(\frac{D}{\Delta x^2}\right)\left(C_{i-1jk} + C_{i+1jk} + C_{ij-1k} + C_{ij+1k} + C_{ijk-1} + C_{ijk+1} - 6C_{ijk}\right) - K_{decay}C_{ijk} \qquad [1]$$

where D is the diffusion coefficient and K_{decay} is the decay rate constant (chemical instability in medium). If the site is occupied by a cell, there is one ODE for the extracellular compartment (eqn 1) and one for the intracellular:

$$\frac{dC_{ijk}^{in}}{dt} = \frac{U'(C_{ijk}, C_{ijk}^{in}, V_{in}) - M'(C_{ijk}^{in}, V_{in})}{V_{in}} - K_{decay}C_{ijk}^{in} \qquad [2]$$

where C^{in} is the intracellular concentration, V_{in} is the cell volume and the mass rate of uptake U' depends on the concentration difference across the cell membrane and the cell surface area.

$$U'(C_{ijk}, C_{ijk}^{in}, V_{in}) \propto (K_{in}C_{ijk} - K_{out}C_{ijk}^{in})V_{in}^{2/3} \qquad [3]$$

The model allows specification of two mass transfer constants to characterize the uptake and efflux rates: K_{in} and K_{out}. For each constituent there is an equation for the mass rate of consumption M', depending on the concentration and, for drugs, on oxygen concentration.

For oxygen, the rate of consumption by metabolism is:

$$M' = \frac{V_{\max} C}{K_m + C} \quad [4]$$

where V_{max} has been determined in HCT116 cells[1] and K_m is assumed to be 1.3 μM (Supplementary Table 4) as previously reported (Secomb et al., 1993).

Oxygen Dependence of Prodrug Metabolism

Hypoxia-activated prodrugs undergo a sequence of reactions in a cell, and the model identifies up to two serial metabolites: Prodrug ➔ Metabolite 1 ➔ Metabolite 2 ➔ untracked products.

The equations that control these three reactions are determined by a set of parameters $K_{met,0}$, F_2, K_{O2}, n, V_{max} and K_m that are specified separately for the parent and each metabolite. The rate constant for the maximum rate of prodrug metabolism, which occurs when oxygen concentration C_{O2} is zero, is $K_{met,0}$.

The actual reaction rate is the product of this rate constant, the intracellular drug concentration, C, and a sigmoid function of oxygen concentration C_{O2}, with parameters F_2, K_{O2} and n:

$$R_i' = \left(1 - F_2 + \frac{F_2 K_{O2}^n}{(K_{O2}^n + C_{O2}^n)} \right) K_{met\,0} C \quad [5]$$

Here F_2 is the fraction of prodrug metabolism that is oxygen dependent, K_{O2} is the oxygen concentration at which oxygen dependent prodrug metabolism is half-maximal and n is the Hill coefficient. With appropriate selection of the parameters, R'_i is the rate of transformation of parent drug (subscript 0 in eqn [6]) to metabolite 1, or of metabolite 1 to metabolite 2, or removal of metabolite 2. The net molar rate functions inserted into (eqn 2) become:

$$\begin{aligned} M_0' &= R_0' \\ M_1' &= R_1' - R_0' \\ M_2' &= R_2' - R_1' \end{aligned} \quad [6]$$

Monolayer ABM

The monolayer model to simulate experiments in monolayer cultures, is based on three simplifying assumptions:

1. Concentrations of all medium constituents depend only on depth (on the z coordinate), i.e. there is no dependence on the lateral position (x,y). This implies a 1D formulation for concentrations.

2. Since all cells are at the same depth, they are all exposed to the same concentrations of nutrients and drugs.

3. The current formulation of cellular metabolism for these cancer cells does not include dependence on cell cycle stage – rates of consumption of oxygen and glucose depend only on the local concentrations of these constituents. Similarly, intracellular drug reaction rates depend only on intracellular oxygen concentration, therefore at any instant all cells have the same intracellular levels of drug and metabolites.

The combination of these assumptions makes it feasible to solve the intracellular reactions for a single cell, using the same parameter values as the spheroid ABM, then use the concentrations for all cells. This leads to a great increase in speed of execution of the model. From the start of the simulation cells are at different points in the cell cycle (through randomization). As a consequence although all cells have the same rates of metabolism, and the same rate of volume growth, cell division and death is not synchronized.

The solvers for oxygen, glucose and for drugs follow a similar pattern. The depth is subdivided into a number of layers (e.g. $NZD = 20$), and the solvers determine the concentrations in each layer, and the intracellular concentrations, in each time step. For oxygen and glucose, the 1D PDE in the medium is converted into a set of ODEs (one for each layer), which is supplemented by the ODE for the intracellular concentration. Mass flux at the lower boundary of the medium grid is given by the rate of uptake of oxygen or glucose by an individual cell multiplied by the number of live cells, and inter-layer mass flux is proportional, through the diffusion coefficient, to the concentration difference. The set of ODEs is solved using the variable time-step Runga-Kutta solver RKC.

In the case of a drug, in general the code allows for three constituents – the parent drug, and two metabolites. The method of solution is the same as for oxygen or glucose, but depending on the drug there can be up to three concentrations in each layer and in the representative cell. Note that drug metabolism reactions occur only within a cell, while instability (first order decay) can occur anywhere. Depending on the relative concentrations in the cell and in the bottom medium layer, the flux of a drug and its metabolites can be either uptake or release.

The upper boundary is a wall (no flux) for glucose and drugs, while in the case of oxygen the concentration has the user-specified fixed value. In each time step, the updating of the constituent concentrations is carried out sequentially, except that in the case of a drug the intracellular reactions for the drug and metabolites are handled together.

Pharmacokinetic/Pharmacodynamic (PKPD) Models

The PKPD models assume that the intrinsic sensitivity of activators and targets to the cytotoxic metabolites is identical. For SN30000 we further assume that Metabolite 1 is a cytotoxic, short-lived free radical (Anderson et al., 2014) that does not diffuse from the cell, while its more stable downstream 1-oxide and nor-oxide metabolites are non-cytotoxic as demonstrated previously (Gu et al., 2017; Hicks et al., 2010; Wang et al., 2014). The PKPD model for SN30000, which has previously been validated in single cell suspensions (Hicks et al., 2006; Hicks et al., 2010), describes the probability of clonogenic cell killing (P_{kill}) in a small time interval Δt, as the product of the killing rate constant K_c, and its rate of bioreductive metabolism M':

$$P_{kill} = K_c M' \Delta t \qquad [7]$$

This formulation assumes the rate of generation of the transient intracellular cytotoxic radical is proportional to M', avoiding the need to calculate explicitly the very low intracellular radical concentrations.

The PR104A model assumes that Metabolites 1 and 2 are DNA interstrand crosslinking cytotoxins with sufficient stability to diffuse from the cell of origin. These metabolites have lifetimes defined by the first order rate constants for their subsequent reactions; the PKPD model is consistent with the reported relationship between PR104A exposure and clonogenic cell killing for single cells (Foehrenbacher et al., 2013):

$$P_{kill} = K_c C \Delta t \quad [8]$$

where C is the intracellular concentration of the PR104A metabolite. When a cell is exposed to two cytotoxic constituents (drug and/or metabolites) at the same time, the probability of survival is given by the product of the two survival probabilities. Therefore, if P_{kill1} and P_{kill2} are the separate kill probabilities for Metabolites 1 and 2, the total probability of cell survival in Δt is

$$P_S = (1 - P_{kill1})(1 - P_{kill2}) \quad [9]$$

and the combined kill probability is $P_{kill} = 1 - P_S$. In each time step, for each cell P_{kill} is calculated based on the intracellular drug and metabolite concentrations. A uniform (0,1) random number R is generated, and if $R < P_{kill}$ the cell is tagged for death. Cell death occurs when the time to divide is reached.

SUPPLEMENTARY FIGURES

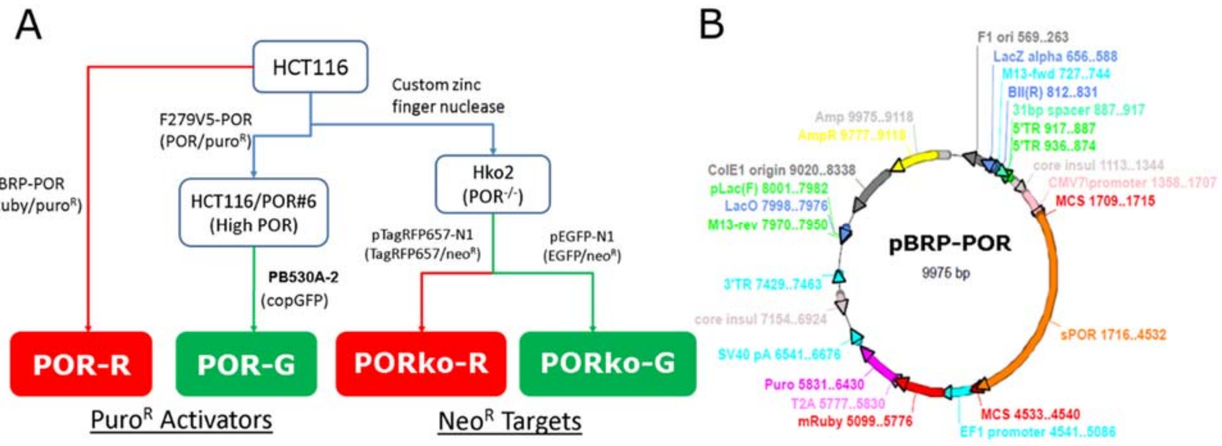

SUPPLEMENTARY FIGURE 1

Development of activator and target cell lines. **(A)** Schematic diagram of generation of activators and targets. To generate red fluorescent activators (POR-R), HCT116 WT cells were transfected with the pBRP-sPOR plasmid using electroporation (Neon™ Transfection System, Invitrogen, Massachusetts, USA) according to the manufacturer's instructions. HCT116/POR#6 cells carrying a puromycin resistance marker (Foehrenbacher et al., 2013) were transfected with a PB514B-2 *piggyBac* plasmid (System Biosciences, California, USA) which encodes copGFP to generate green fluorescent activators (POR-G). Red (PORko-R) or green (PORko-G) fluorescent target cell lines were developed from Hko2 cells in which both *POR* alleles in HCT116 were knocked out with zinc finger nucleases (Su et al., 2013). Hko2 was transfected with the pTagRFP657-N1 plasmid (Addgene) or pEGFP-N1 plasmid (Addgene, Massachusetts, USA), which encode TagRFP657 or EGFP respectively along with neomycin (G418) resistance. As an alternative target cell line, HCT116 cells were transfected with PB713B-1 *piggyBac* plasmid (System Biosciences, California, USA) encoding copGFP and puromycin resistance. Antibiotic selection was applied to cells 2 days after transfection. Cells were allowed to form colonies in 100 mm dishes and individual colonies were transferred into each well in 24-well plates. Two activator and target clones were selected from each transfection based on stability and the level of POR and fluorescent protein marker expression. The resulting cell lines were passaged in selective media as follows: POR-R (high POR, mRuby) and POR-G (high POR, copGFP) in 2 µM puromycin; PORko-R (POR knockout, tagRFP657) and PORko-G (POR knockout, EGFP) in 1mg/ml G418. All cell lines were confirmed *Mycoplasma*-free using PlasmoTest™ (InvivoGen, California, USA). **(B)** Structure of the newly constructed pBRP-POR plasmid. A plasmid for expression of N-terminal truncated POR (sPOR), mRuby fluorescent maker and puromycin resistance (pBRP-POR plasmid) was constructed by ligation of the excised sPOR sequence from the previously reported expression vector F279-V5 (Foehrenbacher et al., 2013) within the multiple cloning site (MCS) of the PB514B-2 *piggyBac* vector (System Biosciences, California, USA). Downstream of POR is an EF1alpha promoter driving the expression of mRuby and puromycin resistance markers. pBRP-POR has key features of the piggyBac transposon system including 5` inverted terminal repeats

(5`TR) for efficient transposition and core insulator elements for stabilized expression. The POR, 5`TR and MCS sequences in pBRP-POR were confirmed by Sanger sequencing.

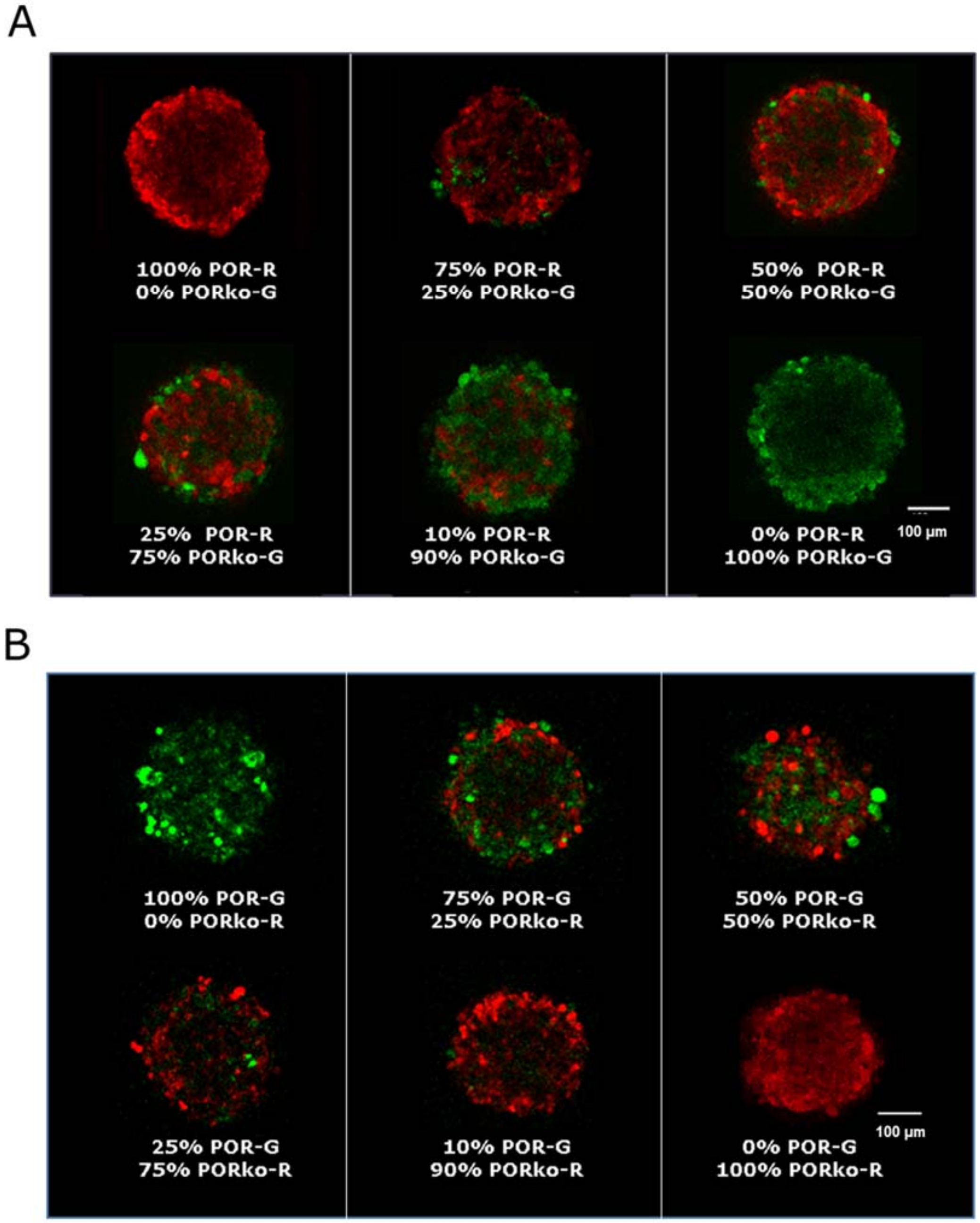

SUPPLEMENTARY FIGURE 2

Distribution of activators and targets in co-culture spheroids. **(A,B)** Confocal images of spheroids grown from a mixture of activators and targets with the indicated initial ratios. Co-culture spheroids composed of POR-R and PORko-G **(A)** or POR-G and PORko-R **(B)** at initial density of 2000 cells/well were grown for 5 days and imaged using a confocal microscope. Scale bar:100 µm.

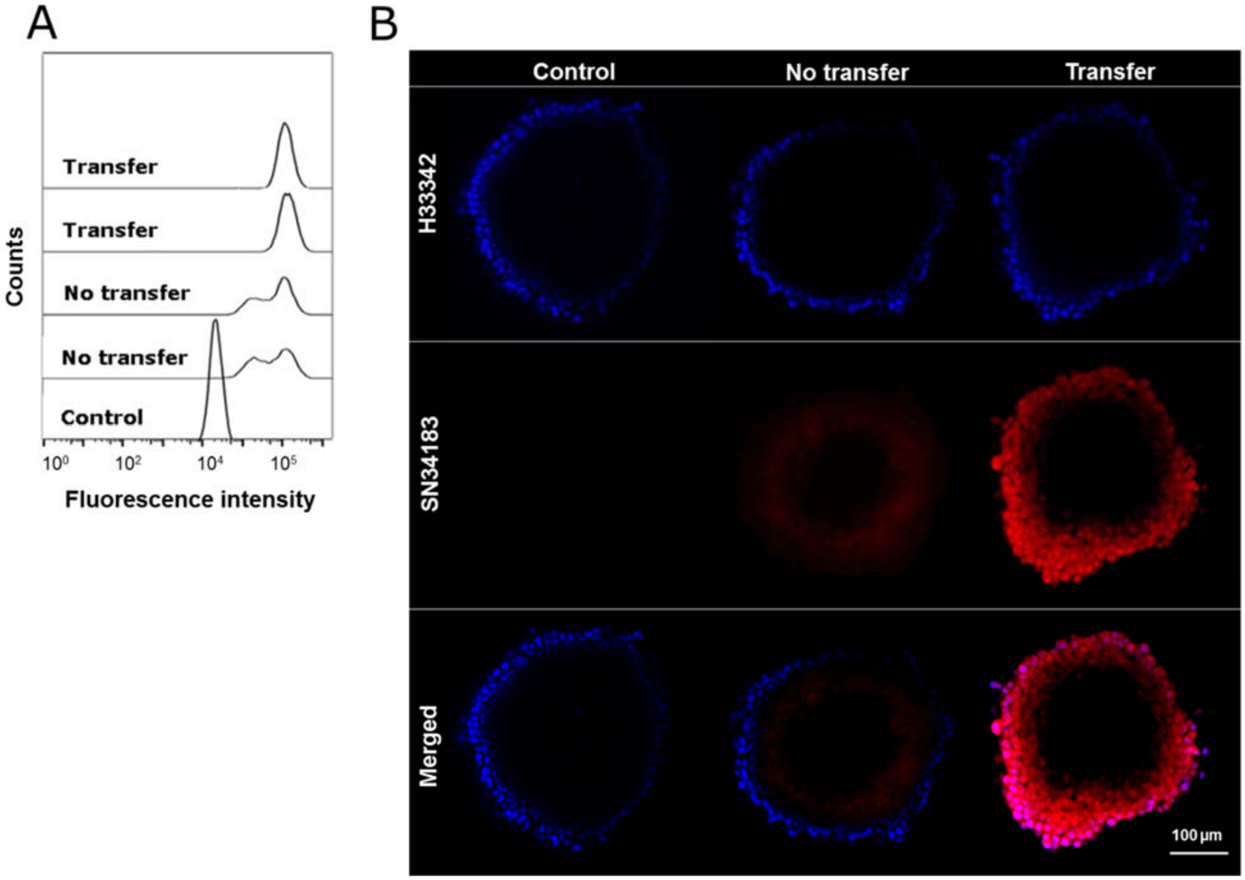

SUPPLEMENTARY FIGURE 3

Deoxygenation of spheroids under anoxia (0% O_2, gas phase). Spheroids were incubated in an anaerobic chamber for 2 h before exposure to a 2-nitroimidazole probe hypoxia probe, SN34183 (2-(2-nitro-1*H*-imidazol-1-yl)-*N*,*N*-di(prop-2-yn-1-yl)acetamide) (Tercel et al., 2011). Spheroids were treated with 100 μM SN34183 either after transferring them to plates that have been pre-equilibrated under anoxia for > 3 days (transfer group) or when spheroids were not transferred to pre-equilibrated plates (no transfer group) for 1 h under anoxia. SN34183 was conjugated with Alexa Fluor 647 azide by click chemistry after fixation with10% neutral-buffered formalin (Tercel et al., 2011). **(A)** Distribution of SN34183 binding determined by flow cytometry (Accuri flow cytometer, BD Biosciences, California, USA) using 488 nm excitation with a 610 nm filter for detection of Alexa 647 fluorescence. Spheroids were pooled and trypsinized prior to flow cytometry analysis. Two separate samples are two pooled spheroids from two different plates. The control is spheroids that were not treated with SN34183 but conjugated with Alexa 647. **(B)** Confocal images of hypoxia staining in control (left), no-transfer (middle) and after-transfer (right) spheroids. A concentration of 8 μM Hoechst33342 was added to spheroids before imaging to stain the surface. Fluorescent images were collected using a Zeiss LSM 710 inverted confocal microscope at 405/410-590 nm (laser/filter) for H33342, 488/661-755 nm for Alexa647. Scale bar represents100 μm, Blue: Hoechst33342, Red: SN34183. The results in (A) and (B) demonstrate that many cells in spheroids are not sufficiently hypoxic to maximally activate SN34183 when the spheroids are not transferred to pre-equilibrated plates.

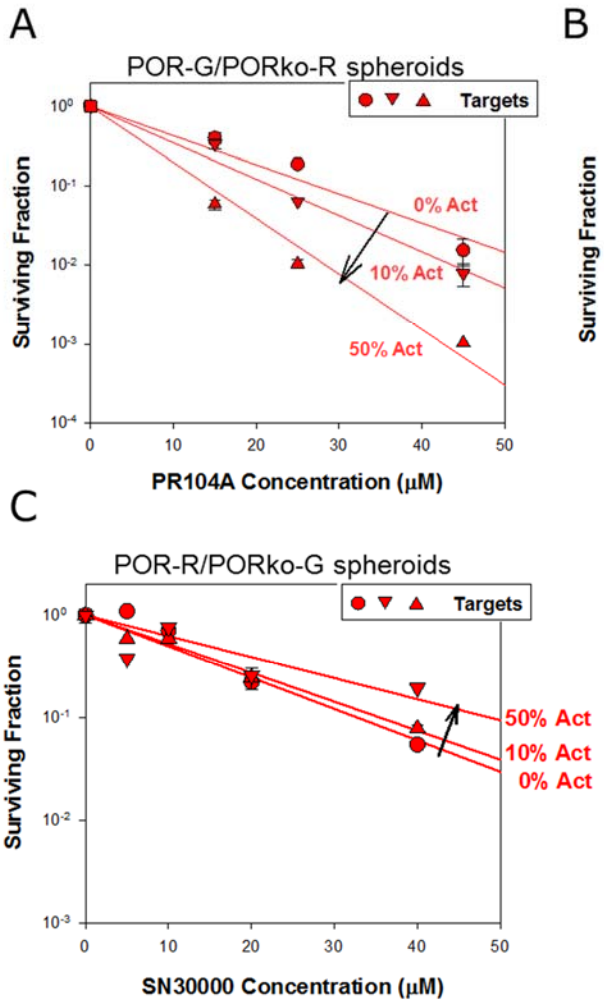

SUPPLEMENTARY FIGURE 4

Investigation of bystander effects in co-culture spheroids under anoxia. **(A)** Clonogenic survival of targets in spheroids initiated with 1000 cells in total, with different ratios of POR-G (activator) and PORko-R (target) cells, and grown for 4 days, giving spheroids smaller than in Figure 3A (average diameter of 309 µm whereas when spheroids were initiated with 3000 cells, average diameter was 429 µm). Spheroids were exposed to PR104A for 1 h under anoxia. **(B)** Clonogenic survival of activators and targets determined from spheroids (seeded at 3000 cells/well, grown for 4 days) comprised of POR-R and PORko-G after exposure to PR104A for 1 h. **(C)** Clonogenic survival of targets in smaller spheroids (1000 cells/well) comprised of POR-R and PORko-G and grown for 4 days after SN30000 treatment for 1 h. Values are means and ranges from 2 biological replicates (4 spheroids/replicate). Lines are regression fits.

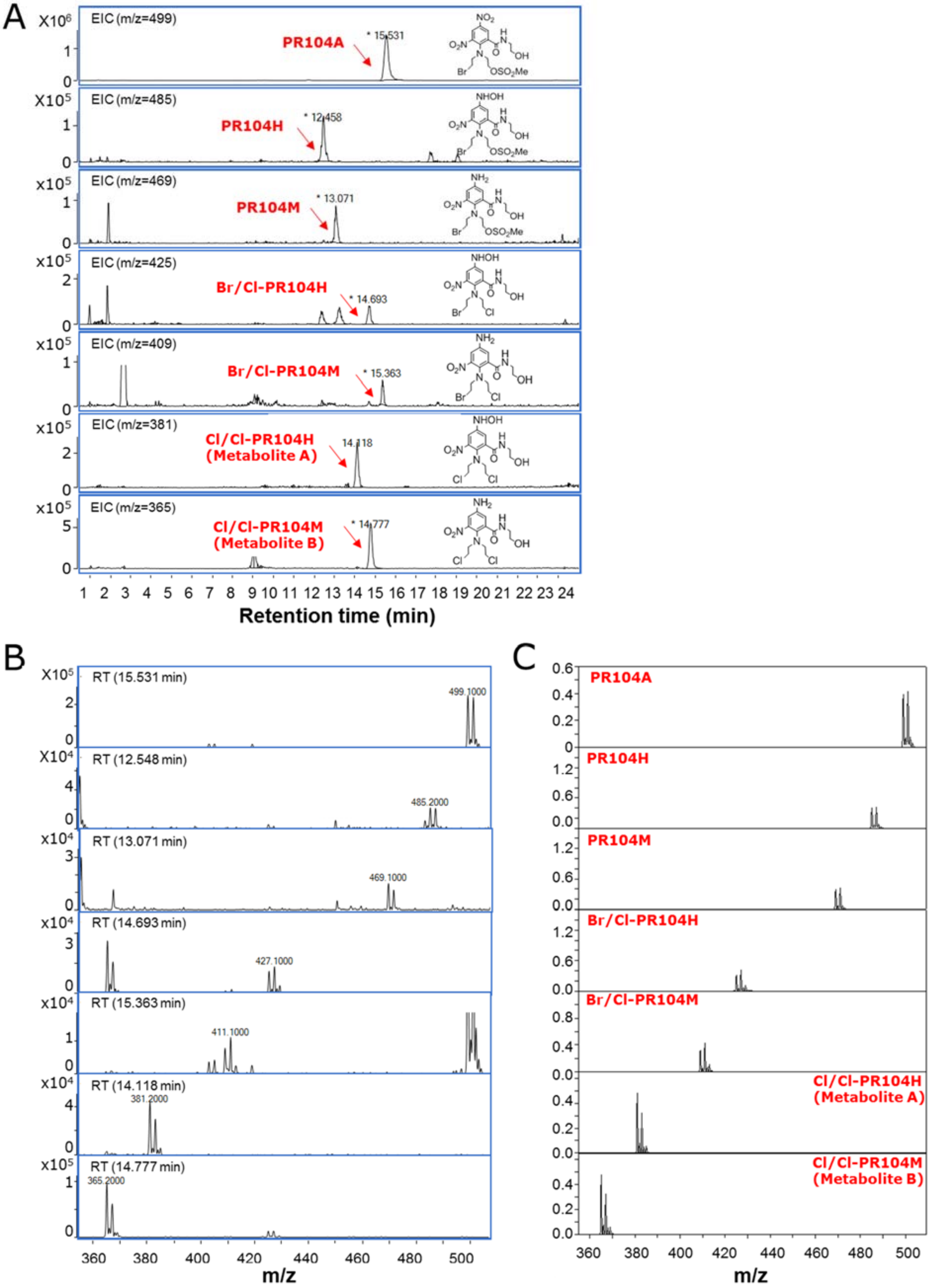
A
EIC (m/z=499)
PR104A
* 15.531
EIC (m/z=485)
PR104H
* 12.458
EIC (m/z=469)
PR104M
* 13.071
EIC (m/z=425)
Br/Cl-PR104H
* 14.693
EIC (m/z=409)
Br/Cl-PR104M
* 15.363
EIC (m/z=381)
Cl/Cl-PR104H
(Metabolite A)
14.118
EIC (m/z=365)
Cl/Cl-PR104M
(Metabolite B)
* 14.777
Retention time (min)
B
RT (15.531 min)
499.1000
RT (12.548 min)
485.2000
RT (13.071 min)
469.1000
RT (14.693 min)
427.1000
RT (15.363 min)
411.1000
RT (14.118 min)
381.2000
RT (14.777 min)
365.2000
m/z
C
PR104A
PR104H
PR104M
Br/Cl-PR104H
Br/Cl-PR104M
Cl/Cl-PR104H
(Metabolite A)
Cl/Cl-PR104M
(Metabolite B)
m/z

SUPPLEMENTARY FIGURE 5

LC-MS/MS analysis of extracellular metabolites of PR104A. **(A,B)** Extracted ion chromatogram of metabolites in POR-R cells exposed to 100 µM PR104A under anoxia for 3 h, demonstrating formation of Metabolite A and B as major chloro derivatives, and the presence of the corresponding Br/Cl intermediates. **(A)** Representative extracted ion chromatograms (EIC). **(B)** Mass spectra of the corresponding species at the indicated retention time of each peak. **(C)** Confirmation of the structural assignment using the theoretical spectra generated using MS-Isotope (http://prospector.ucsf.edu/prospector/cgi-bin/msform.cgi?form=msisotope).

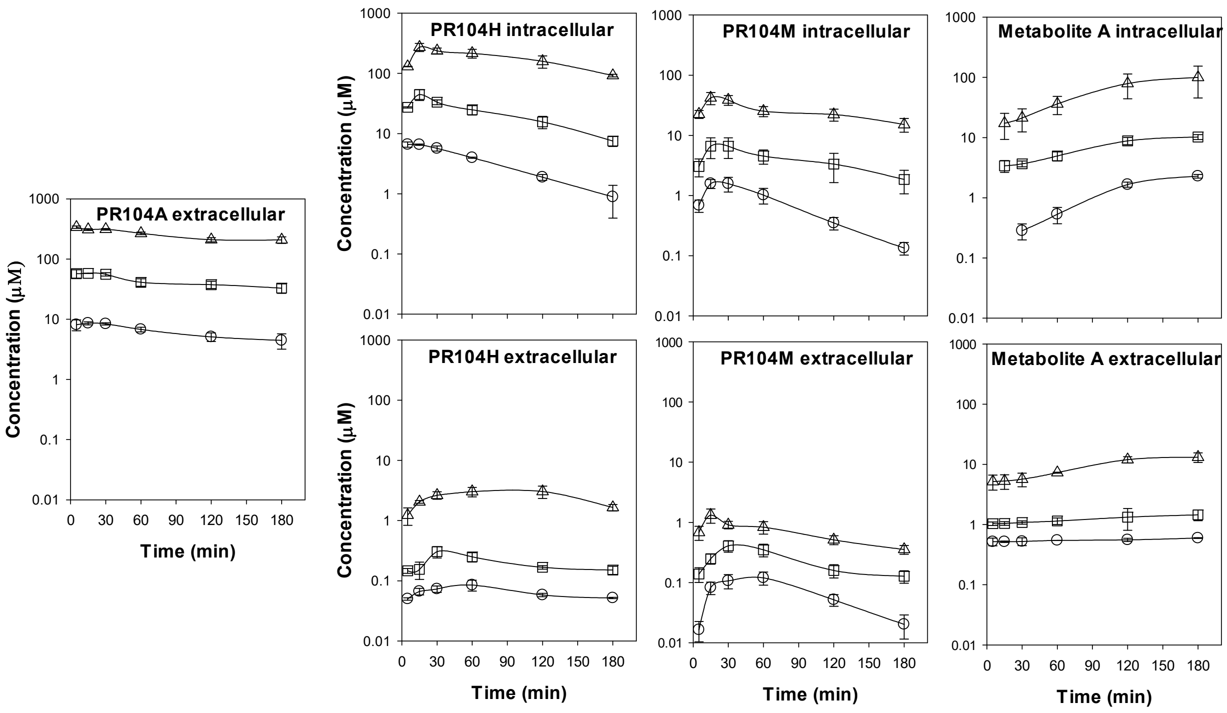

SUPPLEMENTARY FIGURE 6

Concentration-time profile of Metabolite A in SiHa cultures. Intracellular and extracellular concentrations of PR104A and its metabolites in SiHa single cell suspensions ($2x10^6$ cells/mL) treated with 10 µM (o), 60 µM (□) or 300 µM (Δ) PR104A under anoxia after drug was added. PR104A, PR104H and PR104M were quantified using a previously described LC-MS/MS method with stable isotope internal standards (Patel et al., 2007). Metabolite A was quantified using a tetra-deuterated internal standard which was synthesized by zinc dust reduction of tetra-deuterated Cmpd 1 (see Figure 6A). Multiple reaction monitoring (MRM) was used to monitor the mass transitions of Metabolite A and its internal standards with the following parameters: Fragmentation voltage, 134 V; collision energy, 20V; precursor ion, 381 m/z for d_0- and 385 m/z for d_4-metabolite A; product ion, 320 m/z for both d_0 and d_4-metabolite A. Metabolite B (see Figure 6B) was not quantified due to difficulties in preparing pure compound because of low yields following zinc dust reduction of Cmpd 1. Mean ± SE are duplicate determinations at each concentration.

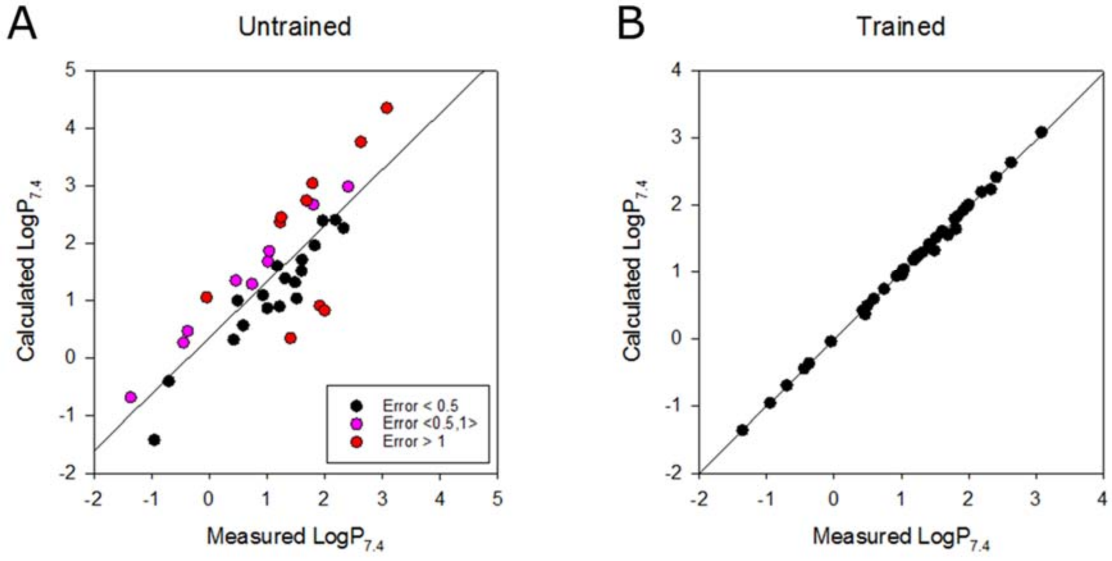

SUPPLEMENTARY FIGURE 7

Measured and calculated LogP values of nitrobenzamide mustards. **(A,B)** Measured LogP values at pH 7.4 of 37 nitrobenzamide mustards and relationship to values calculated using ACD/PhysChem version 12.01 before **(A)** and after **(B)** training the software with a set of 153 measured compounds (including the nitrobenzamide mustards). Octanol-water partition coefficients were measured as described in (Pruijn et al., 2005). Compound structures and individual LogP values are shown in Supplementary Table 5.

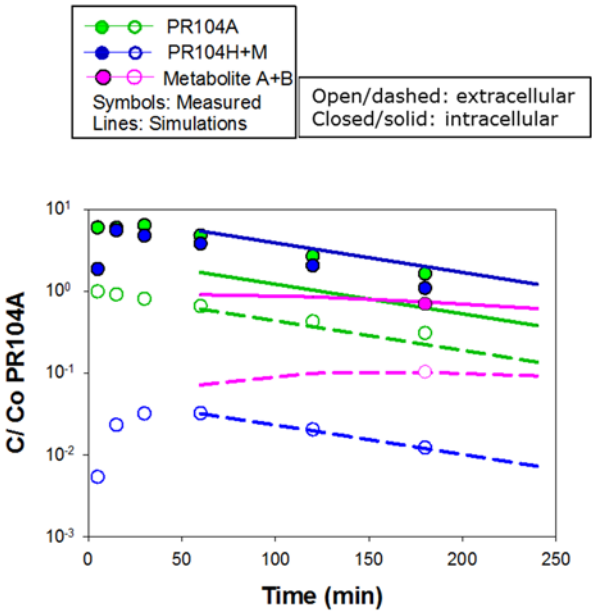

SUPPLEMENTARY FIGURE 8

Development and validation of the 'dichloro metabolites' model. Estimation of cellular PK parameters of Metabolite 1 (PR104H+M) and 2 (Metabolite A+B) (see Figure 6A) using previously measured concentrations of PR104A and PR104H+M in single cell suspensions of HCT116/POR#6 cells (Foehrenbacher et al., 2013). Concentrations are normalized to initial PR104A concentration. $K_{met,0}$ for metabolism of PR104A is unchanged from the published value (Foehrenbacher et al., 2013). Concentrations of Metabolite A+B in single cell suspensions of HCT116/POR#6 were assumed to be same as that in monolayer POR-R cells after 3 h exposure to PR104A (Figure 6C), which is justified by the following observations: (a) Measured intracellular and extracellular concentrations of PR104A and PR104H+M in monolayer POR-R cells (Figure 6C) were in good agreement with those in single cell suspensions of HCT116/POR#6 cells after 3 h exposure to PR104A, and (b) POR-R and HCT116/POR#6 cells showed similar FSL61 reduction (Figure 1B). Parameters values are listed in Supplementary Table 3.

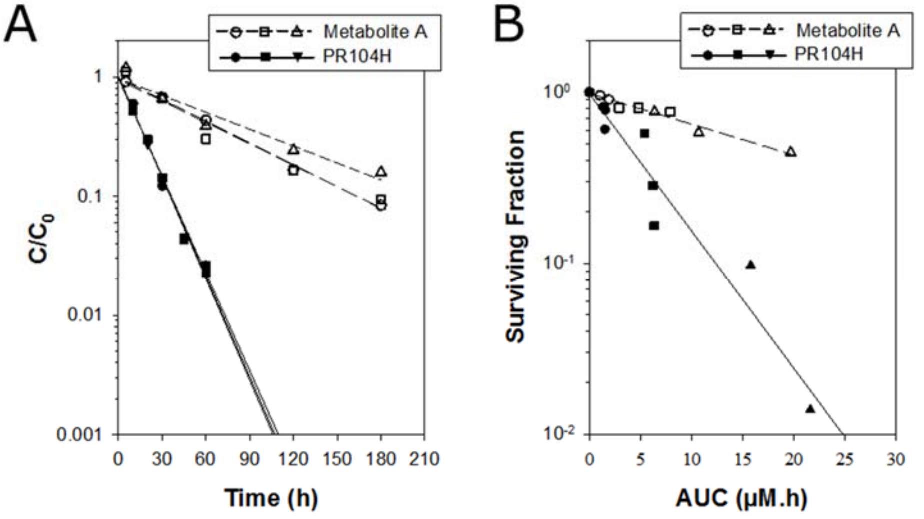

SUPPLEMENTARY FIGURE 9

Concentration-time profile and cytotoxicity of Metabolite A and PR104H in oxic SiHa cell suspensions ($5x10^6$ cells/mL, 20% O_2). (A) Extracellular concentrations of Metabolite A and PR104H over time when Metabolite A or PR104H were added to SiHa cell suspensions. Concentrations are normalized to initial concentrations estimated by extrapolation (initial concentrations: 10 µM (circles), 30 µM (squares) or 100 µM (triangles)). (B) Corresponding clonogenic survival as a function of extracellular AUC, determined by sampling cultures for plating at each timepoint/concentration in (A). AUC values were calculated from the linear transform of the data in (A) by the log trapezoidal rule with extrapolation of the terminal slope to infinity. Lines are linear regressions.

SUPPLEMENTARY TABLES

SUPPLEMENTARY TABLE 1. Population doubling time of parental HCT116, activator and target cell lines, determined in monolayer cultures.

	Doubling time in monolayer (h)[a]
HCT116	20.7 ± 1.5
POR-G	31.9 ±1.0
POR-R	28.7 ± 0.7
PORko-G	23.4 ± 2.3
PORko-R	24.7 ± 0.43

[a] Values are means and SE estimated from the total population increase during 2-3 passages.

SUPPLEMENTARY TABLE 2. SN30000 PKPD parameters

Parameter (unit)	Description	Published value	Reference	ABM parameter
D_S (cm^2s^{-1})	Diffusion coefficient in spheroid	$1.17x10^{-6}$	Mao et al., 2018	$1.17x10^{-6}$
D_M (cm^2s^{-1})	Diffusion coefficient in medium	$7.5x10^{-6}$	Mao et al., 2018	$7.5x10^{-6}$
K_{in} (min^{-1})	Rate constant for transfer from the extracellular to the intracellular compartment	10	Mao et al., 2018	10
K_{out} (min^{-1})	Rate constant for transfer from the intracellular to the extracellular compartment	10	Mao et al., 2018	10
Half-life (h)	Non-enzymatic loss in culture medium at 37 °C.	24000	Mao et al., 2018	24000
Target $K_{met,0}$ (min^{-1})	Rate constant for the maximum rate of metabolism in target cells under anoxia	1.88	Mao et al., 2018	1.3
Activator $K_{met,0}$ (min^{-1})	Rate constant for the maximum rate of metabolism in activator cells under anoxia	-	-	14
F_2	Proportion of O_2-dependent metabolism of the drug (O_2-independent metabolism of the drug = 1- F_2)	1	Hicks et al., 2010	1
K_{O2}	O_2 concentration for half-maximum prodrug activation	1.14	Hicks et al., 2010	1.14
n	Hill parameter n	1.7	Mao et al., 2018	1.7
K_c ($mM^{-1}s^{-1}$)	Intrinsic cytotoxic potency of the prodrug	-	-	1.8

SUPPLEMENTARY TABLE 3. PR104A PKPD parameters

Parameter (unit)	Description	Drugs	Published value	Reference	Dichloro model
D_S (cm^{2}s^{-1})	Diffusion coefficient in spheroid	Parent	1.44×10^{-7}	Foehrenbacher et al., 2013	1.44×10^{-7}
		Metabolite 1	1.44×10^{-7}	Foehrenbacher et al., 2013	1.44×10^{-7}
		Metabolite 2	1.44×10^{-7}	Foehrenbacher et al., 2013	1.44×10^{-7}
D_M (cm^{2}s^{-1})	Diffusion coefficient in medium	Parent	6×10^{-6}	Foehrenbacher et al., 2013	6×10^{-6}
		Metabolite 1	6×10^{-6}	Foehrenbacher et al., 2013	6×10^{-6}
		Metabolite 2	6×10^{-6}	Foehrenbacher et al., 2013	6×10^{-6}
K_{in} (min^{-1})	Rate constant for transfer from the extracellular to the intracellular compartment	Parent	3	Foehrenbacher et al., 2013	3
		Metabolite 1	0.9	Foehrenbacher et al., 2013	0.09
		Metabolite 2	0.9	Foehrenbacher et al., 2013	3
K_{out} (min^{-1})	Rate constant for transfer from the intracellular to the extracellular compartment	Parent	0.5	Foehrenbacher et al., 2013	0.5
		Metabolite 1	0.3	Foehrenbacher et al., 2013	0.09
		Metabolite 2	0.3	Foehrenbacher et al., 2013	0.5
Half-life (h)	Non-enzymatic loss in culture medium at 37 ºC.	Parent	10.2	Foehrenbacher et al., 2013	10.2
		Metabolite 1	0.21	Foehrenbacher et al., 2013	0.15
		Metabolite 2	0.11	Foehrenbacher et al., 2013	1
Target $K_{met,0}$ (min^{-1})	Rate constants for the maximum rate of metabolism in target cells under anoxia	Parent	0.054	Foehrenbacher et al., 2013	0.03
		Metabolite 1	0.9	Foehrenbacher et al., 2013	0.3
		Metabolite 2	0.18	Foehrenbacher et al., 2013	0
Activator $K_{met,0}$ (min^{-1})	Rate constant for the maximum rate of metabolism in activator cells under anoxia	Parent	1.08	Foehrenbacher et al., 2013	2
		Metabolite 1	0.9	Foehrenbacher et al., 2013	0.3

		Metabolite 2	0.18	Foehrenbacher et al., 2013	0
Target F_2	Proportion of O_2-depedent rate of metabolism of the drug in targets (O_2-independent metabolism of the drug = 1- F_2)	Parent	0.977	Foehrenbacher et al., 2013	0.977
		Metabolite 1 and 2	0	Foehrenbacher et al., 2013	0
Activator F_2	Proportion of O_2-depedent rate metabolism of the drug in activators (O_2-independent metabolism of the drug = 1- F_2)	Parent	0.977	Foehrenbacher et al., 2013	0.977
		Metabolite 1 and 2	0	Foehrenbacher et al., 2013	0
K_{O2}	O_2 concentration for half-maximum prodrug activation	Parent	0.126	Hicks et al., 2007	0.126
		Metabolite 1 and 2	n/a[b]	n/a[b]	n/a[b]
n	Hill parameter	Parent	1	Foehrenbacher et al., 2013	2
		Metabolite 1 and 2	1	Foehrenbacher et al., 2013	2
K_c ($mM^{-1}s^{-1}$)	Intrinsic cytotoxic potency	Parent	0	Foehrenbacher et al., 2013	0
		Metabolite 1	0.092	Foehrenbacher et al., 2013	0.092
		Metabolite 2	0.092	Foehrenbacher et al., 2013	0.05

Footnotes:
[a] not O_2 dependent

SUPPLEMENTARY TABLE 4. Oxygen parameters

Parameters (unit)	Description	Value	Reference
D_S (cm^2s^{-1})	Diffusion coefficient in spheroid	$2x10^{-5}$	Grote et al., 1977
D_M (cm^2s^{-1})	Diffusion coefficient in medium	$5x10^{-5}$	Mao et al., 2018
K_{in}, K_{out} (min^{-1})	Rate constants for transfer across the cell membrane	600	Mao et al., 2018
Boundary concentration (mM)	Boundary concentration in medium	0.18	Mao et al., 2018
V_{max} (mol $cell^{-1}s^{-1}$)	Maximum rate of consumption	$6.25x10^{-17}$	Mao et al., 2018
K_m (μM)	Michaelis-Menten constant	1.33	Secomb et al., 1993
n	Hill parameter n	1	Secomb et al., 1993

SUPPLEMENTARY TABLE 5: Measured and calculated octanol:water partition coefficients at pH 7.4 ($LogD_{7.4}$) for a training set of nitrobenzamide mustards

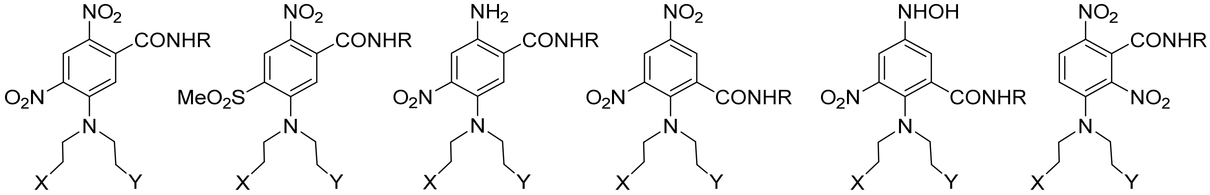

I II III IV V VI

Formula	X	Y	R	Measured $logD_{7.4}$ [a]	Calculated $logD_{7.4}$ untrained [b]	Calculated $logD_{7.4}$ trained [c]
I	Cl	Cl	H	1.515	1.03	1.51
I	OSO_2NH_2	OSO_2NH_2	H	-0.95	-1.42	-0.96
I	Cl	OSO_2CH_3	H	0.425	0.31	0.42
I	Br	OSO_2CH_3	H	0.493	0.99	0.49
I	Iodo	OSO_2CH_3	H	0.743	1.28	0.74
I	Cl	Cl	OH	1.22	0.89	1.22
I	Br	OSO_2CH_3	$CH_2CHOHCH_2OH$	-0.0453	1.05	-0.0454
I	Br	Br	$(CH_2)_3OH$	1.81	2.67	1.64
I	Cl	Cl	CH_2CH_2OH	1.315	1.38	1.29
I	Cl	Br	H	1.61	1.71	1.61
I	Br	Br	H	1.97	2.39	1.97
I	Iodo	Iodo	H	2.408	2.98	2.41
I	OSO_2CH_3	OSO_2CH_3	H	-0.701	-0.41	-0.7
I	Cl	Cl	CH_2CH_2-Nmorp	1.6	1.51	1.6
I	Cl	Cl	$(CH_2)_3OH$	1.49	1.31	1.32
I	Cl	Cl	$CH_2CHOHCH_2OH$	0.93	1.09	0.93
I	Br	Br	$CH_2CHOHCH_2OH$	1.25	2.45	1.25
I	Iodo	Iodo	$CH_2CHOHCH_2OH$	1.79	3.04	1.79
I	Br	Br	CH_2CH_2OH	1.69	2.74	1.55
I	Br	OSO_2CH_3	CH_2CH_2OH	0.461	1.34	0.36
II	Br	Br	H	1.18	1.6	1.18
III	OH	OH	H	-0.445	0.26	-0.45
IV	Br	Br	H	2.63	3.76	2.63
IV	Cl	OSO_2CH_3	H	1.02	1.68	1.02
IV	Br	OSO_2CH_3	H	1.23	2.36	1.23
IV	Br	Br	CH_2CH_2OH	2.33	2.26	2.23

IV	Br	Br	$CH_2CHOHCH_2OH$	1.83	1.96	1.83
IV	Br	OSO_2CH_3	$CH_2CHOHCH_2OH$	0.589	0.56	0.59
IV	Cl	Cl	CH_2CH_2OH	1.92	0.9	1.92
IV	Cl	Cl	$(CH_2)_3OH$	2	0.82	2
IV	Br	OSO_2CH_3	CH_2CH_2OH	1.01	0.86	0.95
IV	Iodo	Iodo	H	3.08	4.35	3.08
V	OH	OH	H	-1.36	-0.69	-1.36
V	Cl	Cl	H	2.19	2.4	2.19
VI	Cl	Cl	H	1.41	0.34	1.41
VI	Br	Br	$CH_2CHOHCH_2OH$	1.04	1.86	1.04
VI	Br	OSO_2CH_3	$CH_2CHOHCH_2OH$	-0.373	0.46	-0.37

Footnotes:

[a] Partition coefficients were measured as described in (Pruijn et al., 2005).

[b] Partition coefficients were calculated using ACD/PhysChem version 12.01 (using ACD/Labs version B).

[c] Partition coefficients were calculated as above using trained data from a User Database that contained 153 different compounds with measured partition coefficients, incl. the 37 nitrobenzamide mustards shown in the table.

REFERENCES

Anderson, R.F., Yadav, P., Patel, D., Reynisson, J., Tipparaju, S.R., Guise, C.P., et al. (2014). Characterisation of radicals formed by the triazine 1,4-dioxide hypoxia-activated prodrug SN30000. Ogr. Biomol. Chem.12, 3386-3392.

Foehrenbacher, A., Patel, K., Abbattista, M., Guise, C.P., Secomb, T.W., Wilson, W.R., et al. (2013). The role of bystander effects in the antitumor activity of the hypoxia-activated prodrug PR-104. Front. Oncol. 3, 263. doi: 10.3389/fonc.2013.00263.

Grote, J., Susskind, R., and Vaupel, P. (1977). Oxygen diffusion constants D and K of tumor tissue (DS-carcinosarcoma) and their temperature dependence. Adv. Exp. Med. Biol. 94, 361-365.

Gu, Y., Chang, T.T.A., Wang, J., Jaiswal, J.K., Edwards, D., Downes, N.J., et al. (2017). Reductive metabolism influences the toxicity and pharmacokinetics of the hypoxia-targeted benzotriazine di-oxide anticancer agent SN30000 in mice. Front. Phar. 8, 531. doi:10.3389/fphar.2017.00531.

Hicks, K.O., Myint, H., Patterson, A.V., Pruijn, F.B., Siim, B.G., Patel, K., et al. (2007). Oxygen dependence and extravascular transport of hypoxia-activated prodrugs: comparison of the dinitrobenzamide mustard PR-104A and tirapazamine. Int. J Radiat. Oncol. Biol. Phys. 69, 560-571.

Hicks, K.O., Pruijn, F.B., Secomb, T.W., Hay, M.P., Hsu, R., Brown, J.M., et al. (2006). Use of three-dimensional tissue cultures to model extravascular transport and predict in vivo activity of hypoxia-targeted anticancer drugs. J Natl. Cancer Inst. 98, 1118-1128.

Hicks, K.O., Siim, B.G., Jaiswal, J.K., Pruijn, F.B., Fraser, A.M., Patel, R., et al. (2010). Pharmacokinetic/pharmacodynamic modeling identifies SN30000 and SN29751 as tirapazamine analogues with improved tissue penetration and hypoxic cell killing in tumors. Clin. Cancer Res. 16, 4946-4957.

Mao, X., Jaiswal, J., McManaway, S., Patel, P., Wilson, W.R., Hicks, K.O., et al. (2018). An agent-based three-dimensional tumour spheroid model that predicts interaction between radiation and hypoxia-activated prodrug SN30000. PLoS Comput. Biol., in press.

Patel, K., Lewiston, D., Gu, Y., Hicks, K.O., and Wilson, W.R. (2007). Analysis of the hypoxia-activated dinitrobenzamide mustard phosphate prodrug PR-104 and its alcohol metabolite PR-104A in plasma and tissues by liquid chromatography-mass spectrometry. J Chromatogr. B. Analyt. Technol. Biomed. Life. Sci. 856, 302-311.

Pruijn, F.B., Sturman, J.R., Liyanage, H.D.S., Hicks, K.O., Hay, M.P., and Wilson, W.R. (2005). Extravascular transport of drugs in tumor tissue: Effect of lipophilicity on diffusion of tirapazamine analogs in multicellular layer cultures. J Med. Chem. 48, 1079-1087.

Secomb, T.W., Hsu, R., Dewhirst, M.W., Klitzman, B., and Gross, J.F. (1993). Analysis of oxygen transport to tumor tissue by microvascular networks. Int. J Radiat. Oncol. Biol. Phys. 25, 481-489.

Su, J., Gu, Y., Pruijn, F.B., Smaill, J.B., Patterson, A.V., Guise, C.P., et al. (2013). Zinc finger nuclease knockout of NADPH:cytochrome P450 oxidoreductase (POR) in human tumour cell lines demonstrates that hypoxia-activated prodrugs differ in POR dependence. J Biol. Chem. 288, 37138-37153.

Tercel, M., and Pruijn, F.B (2011). Agents and methods for detection and/or imaging of hypoxia. N.Z. Patent No PCT/NZ2011/000083(WO/2011/145957). Auckland, N.Z.

Wang, J., Guise, C.P., Dachs, G.U., Phung, Y., Hsu, A.H., Lambie, N.K., et al. (2014). Identification of one-electron reductases that activate both the hypoxia prodrug SN30000 and diagnostic probe EF5. Biochem. Pharmacol. 91, 436-446. doi: 10.1016/j.bcp.2014.08.003.
